# Cryptanalysis and improvement of a biometrics-based authentication and key agreement scheme for multi-server environments

**DOI:** 10.1371/journal.pone.0194093

**Published:** 2018-03-13

**Authors:** Li Yang, Zhiming Zheng

**Affiliations:** 1 Key Laboratory of Mathematics, Informatics and Behavioral Semantics, Ministry of Education, Beihang University, Beijing, China; 2 School of Mathematics and Systems Science, Beihang University, Beijing 100191, China; King Saud University, SAUDI ARABIA

## Abstract

According to advancements in the wireless technologies, study of biometrics-based multi-server authenticated key agreement schemes has acquired a lot of momentum. Recently, Wang et al. presented a three-factor authentication protocol with key agreement and claimed that their scheme was resistant to several prominent attacks. Unfortunately, this paper indicates that their protocol is still vulnerable to the user impersonation attack, privileged insider attack and server spoofing attack. Furthermore, their protocol cannot provide the perfect forward secrecy. As a remedy of these aforementioned problems, we propose a biometrics-based authentication and key agreement scheme for multi-server environments. Compared with various related schemes, our protocol achieves the stronger security and provides more functionality properties. Besides, the proposed protocol shows the satisfactory performances in respect of storage requirement, communication overhead and computational cost. Thus, our protocol is suitable for expert systems and other multi-server architectures. Consequently, the proposed protocol is more appropriate in the distributed networks.

## Introduction

Tremendous advancements in the wireless technologies enhance the quality of on-line services in the distributed networks. It makes plenty of web users enjoy a variety of helpful on-line services in many aspects, for example, on-line work, on-line medicine, on-line shopping and so on [1, 2]. However, there remains a significant problem, namely, how to help web users enjoy so many on-line services while ensuring the confidentiality of their sensitive datas over an insecure channel. Thus, data protection becomes more and more important for every communication participant in the distributed networks. As a remedy, authenticated key establishment protocols are applied for safeguarding the information and defying the threats, which help web users submit their credentials and acquire various on-line services from a number of remote network servers subsequently [3, 4]. Specifically, mutual authentication that makes network servers check the legality of web users and vice-versa minimizes the risk of internet fraud. As a next step, key agreement helps communication participants establish a common session key to ensure their subsequent communication in the open networks [5].

Over the four decades, there are three kinds of typical factors to design an authenticated key establishment protocol, that is, knowledge factor (password), possession factor (smart card) and inherence factor (biometric information), respectively [6–9]. In last few years, Khan [10] presented two biometric-based authentication schemes which possessed the self-authentication and deniability, respectively. In 2013, Kumari and Khan [11] put forward an improved smart card-based authentication protocol with user anonymity for remote users. In recent years, Farash et al. [12] proposed a lightweight authentication scheme which was applied for consumer roaming. Over the last two years, Kumari et al. [13] presented a smart card-based authentication protocol for session initiation service.

More specifically, Lamport [7] put forward the first authentication scheme which was based on password and was unable to provide the key agreement in 1981. However, his protocol maintained some password-verification tables that made stolen verification tables attack feasible. Afterwards, a sequence of improved password-based authentication and key establishment schemes have been presented [14–16]. There are some common shortcomings in these authenticated key exchange protocols which only adopt the password, such as, weak password, dictionary attack, stolen verification tables attack and so on. Thus, it is necessary to add the possession factor to design a novel kind of authenticated key agreement schemes, which makes them more robust [17–19].

Later on, two-factor authentication and key establishment protocols which apply both password and smart card have been deployed widely in the distributed networks. In order to log in the expected remote network servers, web users need to insert their smart card into a smart card reader and enter their password. In 1991, Chang et al. [20] presented a password-based authentication scheme with smart card. Since then, a series of cryptanalysis and improvements have been put forward [21–25]. However, it is practicable to acquire some datas stored in the smart card through side channel attacks [26]. Therefore, a lost or stolen smart card makes authenticated key agreement protocols vulnerable [27–30].

In order to solve these aforementioned problems, biometric information (e.g. facial expressions, retina and finger prints and so on) as an inherence factor has been added to propose a variety of three-factor authenticated key establishment protocols. Different from knowledge factor and possession factor, biometric information which possesses the uniqueness further enhances the security of sensitive datas [31, 32]. Besides, it is exceedingly difficult for adversary to forge the biometrics of web users. Also it does not request web users to remember their biometric information which is hard to be forgotten or lost. Thus, biometric information is combined with both password and smart card mentioned above to make a battery of three-factor authenticated key agreement schemes appear [33–38]. In practice, biometric datas imprinted by web users are not the same each time so that directly adopting them usually results in a low success rate for valid web users [39]. To meet this problem, biometric-based fuzzy extractor which is convenient to be implemented by a smart card is introduced to reduce the failure rate [40]. Besides, Bio-Hash code, namely, user specific code is another way to accommodate this problem [41].

Furthermore, earlier authentication and key establishment protocols are only applied for single-server environments, which don’t consider the applicability of multi-server environments. Specifically, it is inefficient for single-server authentication schemes to be directly adopted in the multi-server environments. With a rapid augmentation of different network servers, web users not only register and login each individual server repeatedly, but also maintain massive credentials about identities and passwords. In 2001, Li et al. [42] put forward the first multi-server authenticated protocol which coped up with this problem mentioned above. In particular, Li et al. [42] efficiently applied a registration center to achieve the single registration in the multi-server architectures. During the past two decades, a large amount of multi-server authentication schemes have been presented, in which some protocols adopt the two-factor [43–46] and others are based on three-factor [47–56].

The multi-server authentication mechanism requires the higher security. Since legal users adopt the same credentials to log into a variety of individual network servers, it is practical for adversaries to make many protocols vulnerable to the user impersonation attack, privileged insider attack and server spoofing attack by tracing web users [47, 57, 58]. As typical multi-server architectures, expert systems which benefit from decision-making capability of human experts have a great deal of applications, for example, security auditing and network management. Particularly, Tsudik and Summers [59] introduced an security auditing expert system called AudES which automated a great deal of manual security auditing procedures in order to alleviate the burden of human auditors. For network management expert systems, Hariri and Jabbour [60] designed a generalized architecture to manage plenty of resources in a distributed computer network. Recently, Mishra et al. [50] put forward an anonymous three-factor multi-server authenticated scheme with key agreement for expert systems which was adopted to ensure the communications between web user and network server. They declared that their protocol provided a high security. However, Wang et al. [61] indicated that Mishra et al.’s scheme was vulnerable to several common attacks and presented an improved protocol to enhance the security. Unfortunately, due to cryptanalysis described below, we claim that Wang et al.’s scheme is still vulnerable to the user impersonation attack, privileged insider attack and server spoofing attack. Besides, their scheme fails to provide the perfect forward secrecy.

As a remedy of these aforementioned problems, we propose a biometric-based authentication and key agreement protocol for multi-server architectures in order to ensure the confidentiality of sensitive datas while web user enjoys some decision-making services, such as security auditing and network management in the expert systems. When web user wants to login the network server to acquire these services, our protocol is performed between web user and network server. Concretely, web user submits his login request message to network server. Next, network server tries to authenticate web user with the message received from web user and the beforehand information saved during the registration phase. Also network server issues his authentication request message to web user. Then, web user tries to authenticate network server in a similar way and delivers his authentication reply to network server. Finally, web user and network server apply our protocol to achieve the mutual authentication and key agreement. Compared with other related schemes, our protocol achieves the stronger security and provides more functionality properties. Besides, the presented protocol requires the lower computational cost and shows a satisfactory performance on the communication overhead with the same level of storage requirement. Thus, the proposed protocol is suitable for expert systems and other multi-server architectures, such as, on-line medicine systems, on-line shopping systems and so on. Above all, our protocol is more appropriate in the distributed networks.

The remaining of this paper is organized in seven sections as below. Next section introduces the collision-resistant hash function, threat assumptions and biometrics-based fuzzy extractor, respectively. Section 3 reviews Wang et al.’s scheme. Section 4 discusses some weaknesses of Wang et al.’s scheme. Section 5 describes the proposed biometrics-based authenticated key agreement protocol in details. And then section 6 provides the security analysis, functionality analysis and efficiency analysis of our protocol, and compares our protocol with others in these aforementioned respects. Last section gives the conclusion.

## Preliminaries

During this section, we briefly describe some concepts relating to collision-resistant hash function, threat assumptions and biometrics-based fuzzy extractor as follows.

### Collision-resistant hash function

According to an arbitrary length binary string, collision-resistant hash function outputs a fixed-length binary string, that is, *h* = *h*(*x*) : 0, 1* → 0, 1^*n*^ [62]. Furthermore, retrieving this arbitrary length input from a given output is computationally infeasible. Thus, collision resistant property is explained as below. For a given input *x*, it is computationally infeasible to find any input *y* ≠ *x* makes *h*(*x*) = *h*(*y*).

### Threat assumptions

During this subsection, we introduce some common threat assumptions which includes the Dolev-Yao threat model [63] and the risk of side-channel attacks [27]. More details about these threat assumptions are described as below.

1. Adversary *E* might be a malicious user or an outside hacker.

2. Adversary *E* has an ability to eavesdrop all communication messages between participants via an open channel.

3. Adversary *E* can modify, delete, resend and reroute all eavesdropped messages.

4. Adversary *E* is able to extract all stored datas from a lost or stolen smart card by examining the power consumption.

### Biometrics-based fuzzy extractor

**Fig 1:**
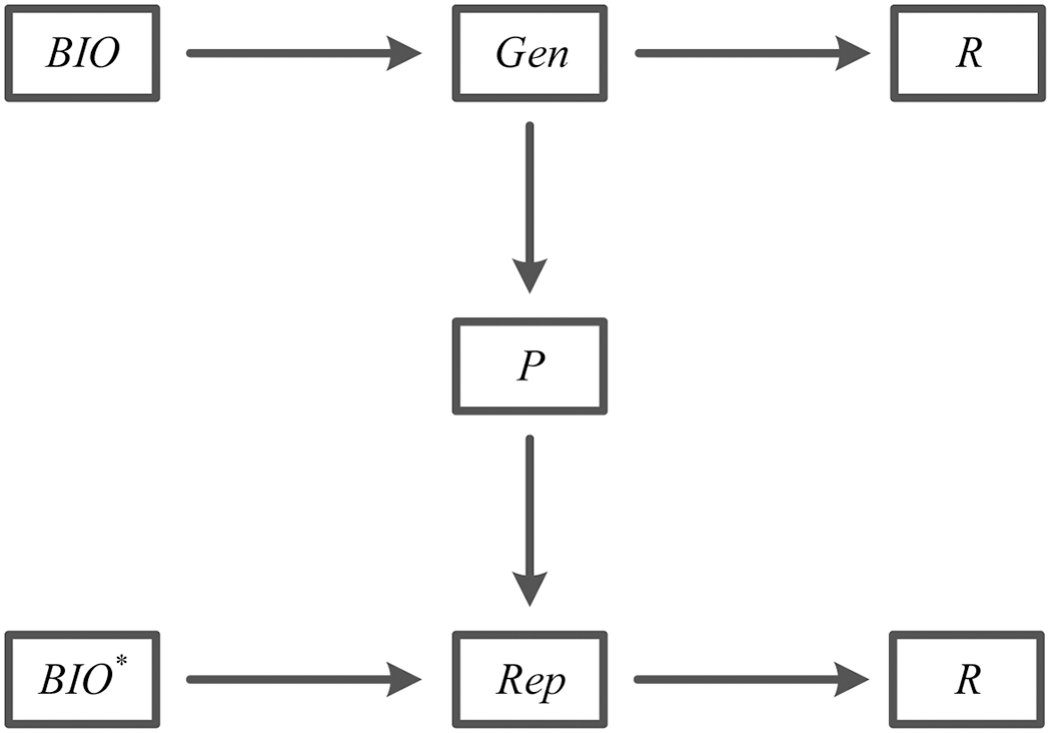
The mechanism of fuzzy extractor.

We briefly introduce the mechanism of biometrics-based fuzzy extractor in this subsection. A biometrics-based fuzzy extractor which converts the biometric information into two available and unpredictable values consist of two procedures, namely, *Gen* and *Rep* [40]. More specifically, details about this mechanism are illustrated in Fig 1. Based on the biometric information *BIO*, procedure *Gen* which is a probabilistic generation function outputs an unpredictable binary string *R* ∈ {0, 1}^*l*^ and an auxiliary binary string *P* ∈ {0, 1}*. With the help of this auxiliary string *P* and another biometric information BIO*, procedure *Rep* which is a deterministic reproduction function recovers a corresponding unpredictable binary string *R*. When *Gen*(*BIO*) → 〈*R*, *P*〉 and *dis*(*BIO*, BIO*) ≤ *t* hold, then we have *Rep*(BIO*, *P*) → *R*. Otherwise, there is no output provided by procedure *Rep*. Furthermore, error-tolerant makes it more robust to recover a corresponding unpredictable binary string *R*, as long as this biometric information BIO* keeps reasonable close to an initial biometrics *BIO*.

Since biometric features vary slightly at every imprint, another way to extract the biometric features is applying the Bio-Hash codes. In recent times, many Bio-Hashing authentication schemes with key agreement are presented [41, 64, 65]. Similarly, Bio-Hashing is also a convenient technique, which is usable in many small devices.

## Review of Wang et al.’s scheme

During this section, we review Wang et al.’s biometrics-based authentication and key agreement scheme for multi-server environments which is described in Ref. [61]. Their scheme includes six phases, namely, server registration phase, user registration phase, login phase, authentication phase, password change phase and user revocation/re-registration phase. There are the following three participants in their scheme, that is, registration center *RC*, server *S*_*j*_ and user *U*_*i*_. Suppose that registration center *RC* is a trusted third party. In Wang et al.’s scheme, registration center *RC* is responsible for user registration and server registration. For convenience, symbols and corresponding notions which are applied in their scheme are respectively shown in Table 1.

**Table 1:** Symbols and corresponding notions in Wang et al.’s scheme.

Symbol	Notion
*RC*	Registration center
*S*_*j*_	*j*th server
*U*_*i*_	*i*th user
*SC*_*i*_	User *U*_*i*_’s smart card
*ID*_*i*_	User *U*_*i*_’s identity
*AID*_*i*_	User *U*_*i*_’s dynamic identity
*PW*_*i*_	User *U*_*i*_’s password
*BIO*_*i*_	User *U*_*i*_’s biometric information
*R*_*i*_	User *U*_*i*_’s unpredictable binary string
*P*_*i*_	User *U*_*i*_’s auxiliary binary string
*SID*_*j*_	Server *S*_*j*_’s identity
*PSK*	Pre shared key
*X*	Master secret key
*h*(⋅)	Collision-resistant hash function
⊕	XOR operation
||	Concatenation operation

### Server registration phase

1. Server *S*_*j*_ submits a join request message to registration center *RC*, which helps server *S*_*j*_ become an authorized server in the expert system.

2. Upon receiving this join request message, registration center *RC* sends server *S*_*j*_ a pre shared key *PSK* to server *S*_*j*_ over a secure channel.

### User registration phase

1. Firstly, user *U*_*i*_ imprints his personal biometric information *BIO*_*i*_ at a sensor. Then sensor sketches *BIO*_*i*_ to extract an unpredictable binary string *R*_*i*_ and an auxiliary binary string *P*_*i*_ from *Gen*(*BIO*_*i*_) → (*R*_*i*_, *P*_*i*_). After that, sensor stores this corresponding auxiliary string *P*_*i*_ in the memory. Next, user *U*_*i*_ enters his identity *ID*_*i*_ and password *PW*_*i*_, and calculates *RPW*_*i*_ = *h*(*PW*_*i*_||*R*_*i*_). Finally, user *U*_*i*_ issues his registration request message {*ID*_*i*_, *RPW*_*i*_} to registration center *RC* through a secure channel.

2. Upon obtaining this registration request message, registration center *RC* adds a novel entry 〈*ID*_*i*_, *N*_*i*_ = 1〉 to an internal database for user *U*_*i*_, in which *N*_*i*_ stands for the times of user registration. And then registration center *RC* successively calculates *A*_*i*_ = *h*(*ID*_*i*_||*x*||*T*_*r*_), *B*_*i*_ = *RPW*_*i*_ ⊕ *h*(*A*_*i*_), *C*_*i*_ = *B*_*i*_ ⊕ *h*(*PSK*), *D*_*i*_ = *PSK* ⊕ *A*_*i*_ ⊕ *h*(*PSK*) and *V*_*i*_ = *h*(*ID*_*i*_||*RPW*_*i*_), where *T*_*r*_ is registration time.

3. Registration center *RC* sends user *U*_*i*_ a smart card *SC*_*i*_ which contains {*B*_*i*_, *C*_*i*_, *D*_*i*_, *V*_*i*_} via a secure channel.

4. After receiving his smart card *SC*_*i*_, user *U*_*i*_ stores his auxiliary string *P*_*i*_ mentioned above into his smart card *SC*_*i*_.

### Login phase

1. User *U*_*i*_ inserts his smart card *SC*_*i*_ into the smart card reader. Then he inputs his identity *ID*_*i*_ and password *PW*_*i*_. Next, user *U*_*i*_ imprints his biometric information $BIO_{i}^{*}$ at a sensor. After that, sensor sketches user *U*_*i*_’s biometric information $BIO_{i}^{*}$ and recovers the unpredictable binary string *R*_*i*_ from $Rep\left(BIO_{i}^{*},P_{i}\right)\to R_{i}$.

2. Smart card *SC*_*i*_ computes *RPW*_*i*_ = *h*(*PW*_*i*_||*R*_*i*_) and checks whether *h*(*ID*_*i*_||*RPW*_*i*_) = *V*_*i*_ is valid. If it is valid, smart card *SC*_*i*_ further computes *h*(*PSK*) = *B*_*i*_ ⊕ *C*_*i*_.

3. Smart card *SC*_*i*_ generates a random number *N*_1_ to calculate *AID*_*i*_ = *ID*_*i*_ ⊕ *h*(*N*_1_), *M*_1_ = *RPW*_*i*_ ⊕ *N*_1_ ⊕ *h*(*PSK*) and *M*_2_ = *h*(*AID*_*i*_||*N*_1_||*RPW*_*i*_||*SID*_*j*_||*T*_*i*_), in which *T*_*i*_ is an additional timestamp.

4. Smart card *SC*_*i*_ delivers user *U*_*i*_’s login request message {*AID*_*i*_, *M*_1_, *M*_2_, *B*_*i*_, *D*_*i*_, *T*_*i*_} to server *S*_*j*_ over an open channel.

### Authentication phase

1. Upon receiving user *U*_*i*_’s login request message, server *S*_*j*_ verifies whether *T*_*i*_ − *T*_*j*_ ≤ Δ*T* holds, in which Δ*T* is a suitable time interval and *T*_*j*_ is the time when server *S*_*j*_ obtains user *U*_*i*_’s login request message. If this verification holds, server *S*_*j*_ continues to execute his next step. Otherwise, user *U*_*i*_’s login request is rejected by server *S*_*j*_.

2. Server *S*_*j*_ retrieves *A*_*i*_ = *D*_*i*_ ⊕ *PSK* ⊕ *h*(*PSK*), *RPW*_*i*_ = *B*_*i*_ ⊕ *h*(*A*_*i*_) and *N*_1_ = *RPW*_*i*_ ⊕ *M*_1_ ⊕ *h*(*PSK*) in order to check whether *h*(*AID*_*i*_||*N*_1_||*RPW*_*i*_||*SID*_*j*_||*T*_*i*_) is consistent with *M*_2_.

3. If it holds, server *S*_*j*_ generates a random number *N*_2_ to calculate their session secret key *SK*_*ij*_ = *h*(*AID*_*i*_||*SID*_*j*_||*N*_1_||*N*_2_).

4. Server *S*_*j*_ computes *M*_3_ = *N*_2_ ⊕ *h*(*AID*_*i*_||*N*_1_) ⊕ *h*(*PSK*) and *M*_4_ = *h*(*SID*_*j*_||*N*_2_||*AID*_*i*_) in order to send his authentication request message {*SID*_*j*_, *M*_3_, *M*_4_} to user *U*_*i*_ through an open channel.

5. After receiving server *S*_*j*_’s authentication request message, smart card *SC*_*i*_ retrieves *N*_2_ = *M*_3_ ⊕ *h*(*AID*_*i*_||*N*_1_) ⊕ *h*(*PSK*) and *SK*_*ij*_ = *h*(*AID*_*i*_||*SID*_*j*_||*N*_1_||*N*_2_) to verify whether *h*(*SID*_*j*_||*N*_2_||*AID*_*i*_) = *M*_4_ holds. If it holds, smart card *SC*_*i*_ calculates *M*_5_ = *h*(*SK*_*ij*_||*N*_1_||*N*_2_) in order to submit user *U*_*i*_’s authentication reply {*M*_5_} to server *S*_*j*_ over an open channel.

6. Server *S*_*j*_ checks whether *h*(*SK*_*ij*_||*N*_1_||*N*_2_) = *M*_5_ is valid. If this verification is valid, server *S*_*j*_ further applies this session key *SK*_*ij*_ to communicate with user *U*_*i*_ in the following communication. Otherwise, authentication phase is rejected by server *S*_*j*_.

### Password change phase

1. User *U*_*i*_ enters his identity *ID*_*i*_ and password *PW*_*i*_, and imprints his biometric information $BIO_{i}^{*}$ at a sensor. After that, sensor sketches user *U*_*i*_’s biometric information $BIO_{i}^{*}$ and recovers the unpredictable binary string *R*_*i*_ from $Rep\left(BIO_{i}^{*},P_{i}\right)\to R_{i}$.

2. Smart card *SC*_*i*_ computes *RPW*_*i*_ = *h*(*PW*_*i*_||*R*_*i*_) and verifies whether *h*(*ID*_*i*_||*RPW*_*i*_) = *V*_*i*_ is valid. If this verification is valid, smart card *SC*_*i*_ asks user *U*_*i*_ for a new password. Otherwise, password change phase is terminated immediately by smart card *SC*_*i*_.

3. User *U*_*i*_ enters his new password $PW_{i}^{new}$ and smart card *SC*_*i*_ further calculates $RPW_{i}^{new}=h(PW_{i}^{new}\left|\right|R_{i})$, $B_{i}^{new}=B_{i}⊕RPW_{i}⊕RPW_{i}^{new}$, $C_{i}^{new}=C_{i}⊕RPW_{i}⊕RPW_{i}^{new}$ and $V_{i}^{new}=h(ID_{i}||RPW_{i}^{new})$.

4. In the memory, smart card *SC*_*i*_ respectively replaces *B*_*i*_ with $B_{i}^{new}$, *C*_*i*_ with $C_{i}^{new}$ and *V*_*i*_ with $V_{i}^{new}$.

### User revocation/re-registration phase

1. When user *U*_*i*_ wants to revoke his privilege, he submits a revocation request message, his smart card *SC*_*i*_ and verification message {*RPW*_*i*_} to registration center *RC* via a secure channel. Registration center *RC* checks whether user *U*_*i*_ is valid. If user *U*_*i*_ is valid, registration center *RC* further modifies a corresponding entry by setting 〈*ID*_*i*_, *N*_*i*_ = 0〉.

2. Similarly, after receiving a re-registration request message through a secure channel, registration center *RC* performs these steps mentioned in the subsection 3.2 and replaces 〈*ID*_*i*_, *N*_*i*_ = *N*_*i*_ + 1〉 with 〈*ID*_*i*_, *N*_*i*_〉 to help user *U*_*i*_ re-register.

## Cryptanalysis of Wang et al.’s scheme

In this section, we propose a cryptanalysis of Wang et al.’s scheme. In particular, results demonstrate that their protocol is still vulnerable to the user impersonation attack, privileged insider attack and server spoofing attack. Furthermore, their scheme fails to achieve the perfect forward secrecy. More details of these problems are shown in the following subsections.

### User impersonation attack

Suppose that adversary *E* is an outside hacker who steals user *U*_*i*_’s smart card *SC*_*i*_ and eavesdrops all communications between user *U*_*i*_ and server *S*_*j*_. Specifically, adversary *E* has an ability to extract the stored datas {*B*_*i*_, *C*_*i*_, *D*_*i*_, *V*_*i*_, *P*_*i*_} from user *U*_*i*_’s smart card *SC*_*i*_ by side-channel attacks. Also he is able to collect user *U*_*i*_’s login request message {*AID*_*i*_, *M*_1_, *M*_2_, *B*_*i*_, *D*_*i*_, *T*_*i*_}. Thus Wang et al.’s scheme is vulnerable to user impersonation attack. More narrowly, adversary *E* can impersonate as a legal user so that he is authenticated by server *S*_*j*_. More details are explained as below.

1. Firstly, adversary *E* computes *h*(*PSK*) = *B*_*i*_ ⊕ *C*_*i*_. Then he generates a random number $N_{1}^{*}$ and further calculates $B_{i}^{*}=B_{i}⊕h\left(PSK\right)$, $D_{i}^{*}=h\left(PSK\right)$, $M_{1}^{*}=B_{i}⊕N_{1}^{*}⊕h\left(PSK\right)$ and $M_{2}^{*}=h(AID_{i}\left|\right|N_{1}^{*}\left|\right|B_{i}||SID_{j}\left|\right|T_{i}^{*})$, in which $T_{i}^{*}$ is a current timestamp. Finally, adversary *E* delivers his login request message $\left\{AID_{i},M_{1}^{*},M_{2}^{*},B_{i}^{*},D_{i}^{*},T_{i}^{*}\right\}$ to server *S*_*j*_ over an open channel.

2. When obtaining this login request message from adversary *E*, server *S*_*j*_ verifies whether $T_{i}^{*}-T_{j}^{*}\leq \Delta T$ holds, where $T_{j}^{*}$ is the time when server *S*_*j*_ receives adversary *E*’s login request message. Thus adversary *E* passes server *S*_*j*_’s verification successfully and server *S*_*j*_ continues to execute the subsequent steps normally.

3. Server *S*_*j*_ retrieves $A_{i}=D_{i}^{*}⊕PSK⊕h\left(PSK\right)$, $RPW_{i}=B_{i}^{*}⊕h\left(A_{i}\right)=B_{i}$ and $N_{1}=RPW_{i}⊕M_{1}^{*}⊕h\left(PSK\right)=N_{1}^{*}$ to check whether $h(AID_{i}\left|\right|N_{1}||RPW_{i}||SID_{j}\left|\right|T_{i}^{*})=M_{2}^{*}$ holds. Next server *S*_*j*_ generates a random number $N_{2}^{*}$ and further calculate $SK_{ij}^{*}=h(AID_{i}||SID_{j}\left|\right|N_{1}^{*}\left|\right|N_{2}^{*})$, $M_{3}^{*}=N_{2}^{*}⊕h(AID_{i}\left|\right|N_{1}^{*})⊕h\left(PSK\right)$ and $M_{4}^{*}=h(SID_{j}\left|\right|N_{2}^{*}||AID_{i})$. Lastly, server *S*_*j*_ sends his authentication request message $\left\{SID_{j},M_{3}^{*},M_{4}^{*}\right\}$ to adversary *E* through an open channel as usual.

4. Upon receiving server *S*_*j*_’s authentication request message, adversary *E* retrieves $N_{2}^{*}=M_{3}^{*}⊕h(AID_{i}\left|\right|N_{1}^{*})⊕h\left(PSK\right)$ and $SK_{ij}^{*}=h(AID_{i}||SID_{j}\left|\right|N_{1}^{*}\left|\right|N_{2}^{*})$ in order to calculate $M_{5}^{*}=h(SK_{ij}^{*}\left|\right|N_{1}^{*}\left|\right|N_{2}^{*})$ and submit his authentication reply $\left\{M_{5}^{*}\right\}$ to server *S*_*j*_.

5. Server *S*_*j*_ checks whether $h(SK_{ij}^{*}\left|\right|N_{1}^{*}\left|\right|N_{2}^{*})=M_{5}^{*}$ is valid.

Thus server *S*_*j*_ authenticates adversary *E* and they both apply the session key $SK_{ij}^{*}$ in the following communication. Unfortunately, server *S*_*j*_ mistakenly believes that he communicates with user *U*_*i*_. Therefore Wang et al.’s scheme becomes vulnerable to the user impersonation attack.

### Privileged insider attack

As shown in this subsection, adversary *E* who is a privileged insider can impersonate as user *U*_*i*_ if he steals user *U*_*i*_’s smart card *SC*_*i*_ and eavesdrops all communications between user *U*_*i*_ and registration center *RC*. Similarly, adversary *E* is able to acquire these datas {*B*_*i*_, *C*_*i*_, *D*_*i*_, *V*_*i*_, *P*_*i*_} from smart card *SC*_*i*_. And he has an ability to collect user *U*_*i*_’s registration request message {*ID*_*i*_, *RPW*_*i*_}. So Wang et al.’s scheme is also vulnerable to the privileged insider attack. More details are described as follows.

1. Firstly, adversary *E* computes *h*(*PSK*) = *B*_*i*_ ⊕ *C*_*i*_ and generates a random number *N*_1*E*_. Then he calculates *AID*_*iE*_ = *ID*_*i*_ ⊕ *h*(*N*_1*E*_), *M*_1*E*_ = *RPW*_*i*_ ⊕ *N*_1*E*_ ⊕ *h*(*PSK*) and *M*_2*E*_ = *h*(*AID*_*iE*_||*N*_1*E*_||*RPW*_*i*_||*SID*_*j*_||*T*_*iE*_), where *T*_*iE*_ is a current timestamp. Lastly, adversary *E* issues his login request message {*AID*_*iE*_, *M*_1*E*_, *M*_2*E*_, *B*_*i*_, *D*_*i*_, *T*_*iE*_} to server *S*_*j*_ over an open channel.

2. After acquiring this login request message, server *S*_*j*_ verifies whether *T*_*iE*_ − *T*_*jE*_ ≤ Δ*T* holds, where *T*_*jE*_ is the time when server *S*_*j*_ acquire adversary *E*’s login request message. Unfortunately, adversary *E*’s verification is valid.

3. Server *S*_*j*_ retrieves *A*_*i*_ = *D*_*i*_ ⊕ *PSK* ⊕ *h*(*PSK*), *RPW*_*i*_ = *B*_*i*_ ⊕ *h*(*A*_*i*_) and *N*_1*E*_ = *RPW*_*i*_ ⊕ *M*_1*E*_ ⊕ *h*(*PSK*) in order to verify whether *h*(*AID*_*iE*_||*N*_1*E*_||*RPW*_*i*_||*SID*_*j*_||*T*_*iE*_) is consistent with *M*_2*E*_. Then server *S*_*j*_ generates a random number *N*_2*E*_ and further calculates *SK*_*ijE*_ = *h*(*AID*_*iE*_||*SID*_*j*_||*N*_1*E*_||*N*_2*E*_), *M*_3*E*_ = *N*_2*E*_ ⊕ *h*(*AID*_*iE*_||*N*_1*E*_) ⊕ *h*(*PSK*) and *M*_4*E*_ = *h*(*SID*_*j*_||*N*_2*E*_||*AID*_*iE*_). Finally, server *S*_*j*_ submits his authentication request message {*SID*_*j*_, *M*_3*E*_, *M*_4*E*_} to adversary *E* via an open channel without any suspicion.

4. When receiving server *S*_*j*_’s authentication request message, adversary *E* retrieves *N*_2*E*_ = *M*_3*E*_ ⊕ *h*(*AID*_*iE*_||*N*_1*E*_) ⊕ *h*(*PSK*) and *SK*_*ijE*_ = *h*(*AID*_*iE*_||*SID*_*j*_||*N*_1*E*_||*N*_2*E*_). Then he calculates *M*_5*E*_ = *h*(*SK*_*ijE*_||*N*_1*E*_||*N*_2*E*_) and sends his authentication reply {*M*_5*E*_} to server *S*_*j*_.

5. Server *S*_*j*_ checks whether *h*(*SK*_*ijE*_||*N*_1*E*_||*N*_2*E*_) = *M*_5*E*_ holds as usual.

So server *S*_*j*_ further applies the session key *SK*_*ijE*_ to communicate with adversary *E* and authenticates adversary *E* who is a privileged insider and impersonates as user *U*_*i*_. Unfortunately, Wang et al.’s scheme is unable to resist the privileged insider attack.

### Server spoofing attack

In this subsection, we suppose that adversary *E* who is an insider but isn’t another server *S*_*k*_ has an ability to eavesdrop user *U*_*i*_’s registration request message {*ID*_*i*_, *RPW*_*i*_} and steal user *U*_*i*_’s smart card *SC*_*i*_. Furthermore, adversary *E* is able to collect some datas, for example, {*B*_*i*_, *C*_*i*_, *D*_*i*_, *V*_*i*_, *P*_*i*_}. Thus adversary *E* can masquerade as server *S*_*j*_ to cheat user *U*_*i*_. Therefore Wang et al.’s scheme becomes vulnerable to the server spoofing attack. More details are shown as below.

1. Firstly, adversary *E* calculates *h*(*PSK*) = *B*_*i*_ ⊕ *C*_*i*_ and eavesdrops user *U*_*i*_’s login request message {*AID*_*i*_, *M*_1_, *M*_2_, *B*_*i*_, *D*_*i*_, *T*_*i*_}.

2. Secondly, adversary *E* computes *N*_1_ = *RPW*_*i*_ ⊕ *M*_1_ ⊕ *h*(*PSK*) and generates a fresh random number $N_{2}^{E}$.

3. Next adversary *E* further computes $M_{3}^{E}=N_{2}^{E}⊕h(AID_{i}\left|\right|N_{1})⊕h\left(PSK\right)$ and $M_{4}^{E}=h(SID_{j}\left|\right|N_{2}^{E}||AID_{i})$.

4. Finally adversary *E* issues his authentication request message $\left\{SID_{j},M_{3}^{E},M_{4}^{E}\right\}$ to user *U*_*i*_ over a public channel.

Furthermore, this fake authentication request message is successfully checked. Particularly, adversary *E* is treated as server *S*_*j*_ by user *U*_*i*_ without any doubt. In conclusion, Wang et al.’s scheme can’t resist the server spoofing attack.

### No perfect forward secrecy

During this subsection, we point out that Wang et al.’s scheme does not possess the perfect forward secrecy. Suppose that adversary *E* is a privileged insider who eavesdrops user *U*_*i*_’s registration request message {*ID*_*i*_, *RPW*_*i*_} and steals user *U*_*i*_’s smart card *SC*_*i*_. Particularly, adversary *E* can extract these datas which include *B*_*i*_, *C*_*i*_, *D*_*i*_, *V*_*i*_ and *P*_*i*_ from smart card *SC*_*i*_. More details are described as follows.

1. Firstly, adversary *E* computes *h*(*PSK*) = *B*_*i*_ ⊕ *C*_*i*_ and collects user *U*_*i*_’s login request message {*AID*_*i*_, *M*_1_, *M*_2_, *B*_*i*_, *D*_*i*_, *T*_*i*_}.

2. Secondly, adversary *E* calculates *N*_1_ = *RPW*_*i*_ ⊕ *M*_1_ ⊕ *h*(*PSK*) and further collects server *S*_*j*_’s authentication request message $\left\{SID_{j},M_{3}^{E},M_{4}^{E}\right\}$.

3. Finally adversary *E* computes *N*_2_ = *M*_3_ ⊕ *h*(*AID*_*i*_||*N*_1_) ⊕ *h*(*PSK*) in order to retrieve *SK*_*ij*_ = *h*(*AID*_*i*_||*SID*_*j*_||*N*_1_||*N*_2_).

Therefore it is demonstrated that Wang et al.’s scheme is unable to achieve the perfect forward secrecy.

## The proposed scheme

During this section, we propose a novel biometrics-based authentication and key agreement scheme for multi-server environments which is based on cryptanalysis of Wang et al.’s scheme. Our protocol is built by applying the collision-resistant hash function, EOR operation and concatenation operation. The presented scheme consists of six phases, namely, server registration phase, user registration phase, login phase, authentication phase, password change phase and user revocation/re-registration phase. And there are three participants in our algorithm, that is, registration center *RC*, server *S*_*j*_ and user *U*_*i*_. In our protocol, server *S*_*j*_ and user *U*_*i*_ are able to join the network by registering with registration center *RC*. Besides, mutual authentication only carries out between server *S*_*j*_ and user *U*_*i*_ without intervening registration center *RC*. For convenience, symbols and corresponding notions which are applied in our scheme are respectively shown in Table 2.

**Table 2:** Symbols and corresponding notions in our scheme.

Symbol	Notion
*RC*	Registration center
*S*_*j*_	*j*th server
*U*_*i*_	*i*th user
*SC*_*i*_	User *U*_*i*_’s smart card
*ID*_*i*_	User *U*_*i*_’s identity
*PW*_*i*_	User *U*_*i*_’s password
*BIO*_*i*_	User *U*_*i*_’s biometric information
*R*_*i*_	User *U*_*i*_’s unpredictable binary string
*P*_*i*_	User *U*_*i*_’s auxiliary binary string
*SID*_*j*_	Server *S*_*j*_’s identity
*PSK*	Pre shared key
*s*	Master secret key
*h*(⋅)	Collision-resistant hash function
⊕	XOR operation
||	Concatenation operation

In particular, our proposed scheme enhances Wang et al.’s scheme in these aspects: 1) it resists the user impersonation attack, 2) it prevents the privileged insider attack, 3) it is secure against the server spoofing attack and 4) it provides the perfect forward secrecy. More details are described in these following subsections.

### Server registration phase

**Fig 2:**
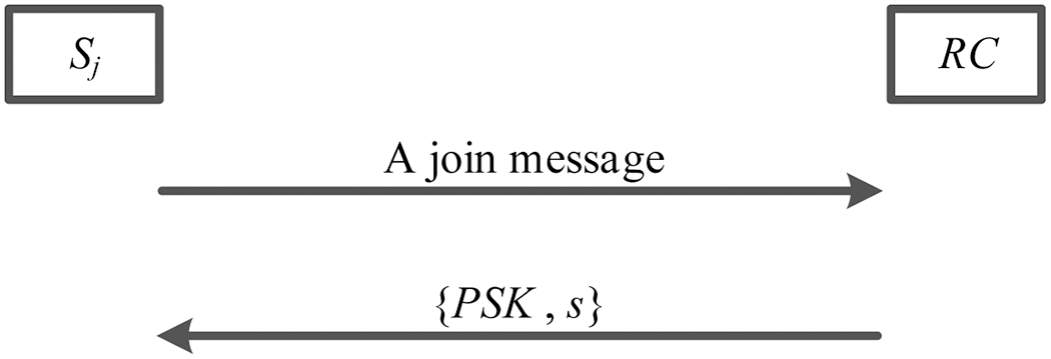
The server registration phase.

New server *S*_*j*_ needs to execute the server registration phase with registration center *RC* through a secure channel. More specifically, server registration phase of the proposed scheme is shown in the Fig 2 and details are described as below.

1. If it wants to be an authorized server in the multi-server environment, server *S*_*j*_ issues a join request message to registration center *RC*.

2. When obtaining this join request message, registration center *RC* authorizes server *S*_*j*_ and replies with a pre shared key *PSK* and a master secret key *s* to server *S*_*j*_ by applying the Key Exchange Protocol (IKEv2) via a secure channel.

3. After receiving a pre shared key *PSK* and a master secret key *s*, authorized server *S*_*j*_ adopts these shared datas, such as *PSK* and *h*(*PSK*), to verify user *U*_*i*_’s legitimacy in the authentication phase.

### User registration phase

**Fig 3:**
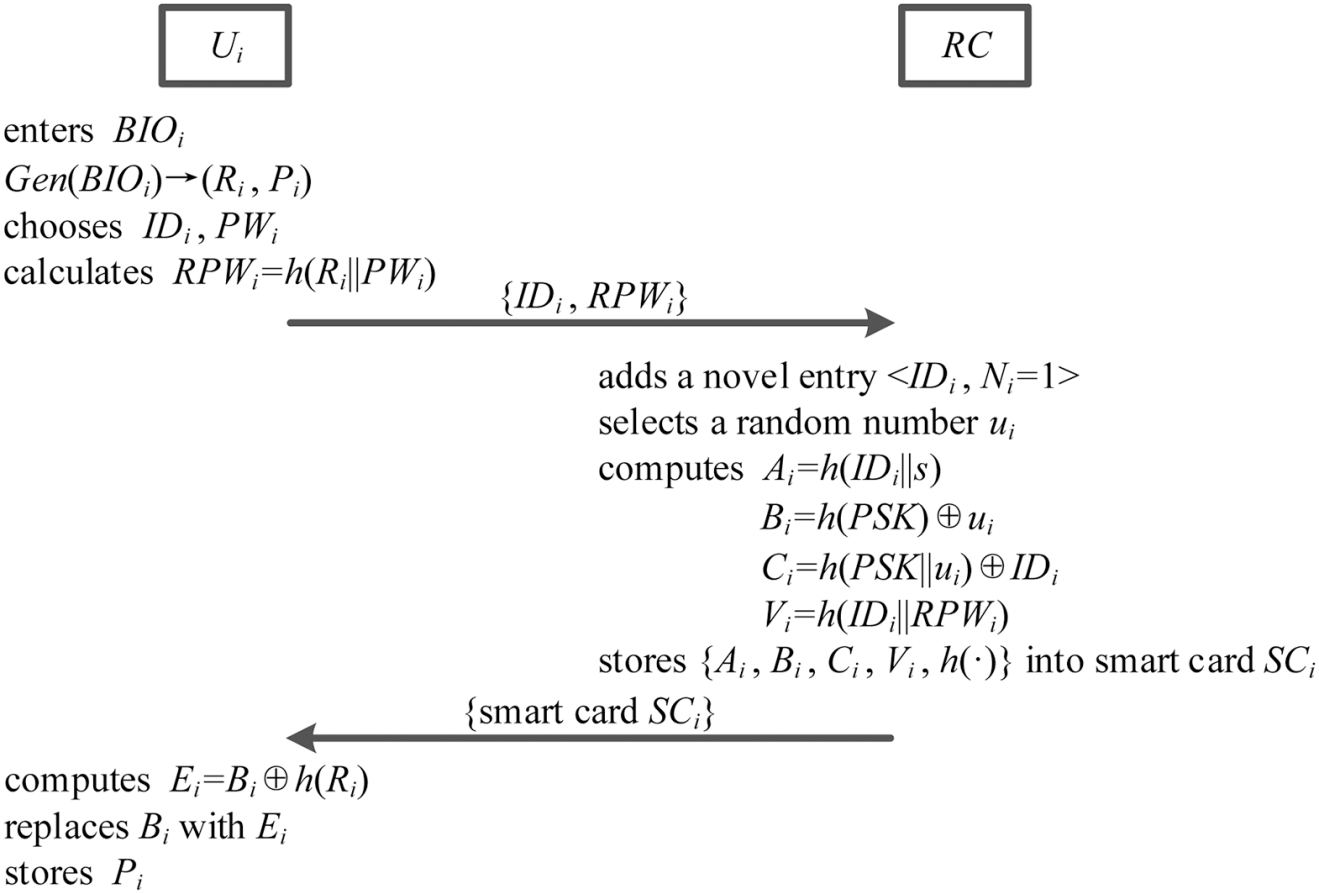
The user registration phase.

New user *U*_*i*_ should perform the user registration phase with registration center *RC* over a secure channel. As details, user registration phase of ours is illustrated in the Fig 3 and explained as follows.

1. Firstly, user *U*_*i*_ enters his personal biometric information *BIO*_*i*_ at a sensor. And then, sensor sketches user *U*_*i*_’s biometrics *BIO*_*i*_, extracts (*R*_*i*_, *P*_*i*_) from *Gen*(*BIO*_*i*_) → (*R*_*i*_, *P*_*i*_), and stores user *U*_*i*_’s auxiliary binary string *P*_*i*_ in the memory. Next, user *U*_*i*_ chooses his identity *ID*_*i*_ and password *PW*_*i*_, and calculates *RPW*_*i*_ = *h*(*R*_*i*_||*PW*_*i*_). Finally, user *U*_*i*_ submits his registration request message {*ID*_*i*_, *RPW*_*i*_} to registration center *RC* through a secure channel.

2. Upon obtaining this registration request message, registration center *RC* adds a novel entry 〈*ID*_*i*_, *N*_*i*_ = 1〉 to his internal database, in which *N*_*i*_ denotes the times of user registration for user *U*_*i*_. Then registration center *RC* selects a random number *u*_*i*_, and calculates *A*_*i*_ = *h*(*ID*_*i*_||*s*), *B*_*i*_ = *h*(*PSK*) ⊕ *u*_*i*_, *C*_*i*_ = *h*(*PSK*||*u*_*i*_) ⊕ *ID*_*i*_ and *V*_*i*_ = *h*(*ID*_*i*_||*RPW*_*i*_).

3. Registration center *RC* sends user *U*_*i*_’s smart card *SC*_*i*_ which includes {*A*_*i*_, *B*_*i*_, *C*_*i*_, *V*_*i*_, *h*(⋅)} via a secure channel.

4. After receiving this smart card *SC*_*i*_, user *U*_*i*_ computes *E*_*i*_ = *B*_*i*_ ⊕ *h*(*R*_*i*_) and replaces *B*_*i*_ with *E*_*i*_. Finally, *U*_*i*_ stores his auxiliary binary string *P*_*i*_ into his smart card *SC*_*i*_, and initializes the login and authentication environments.

### Login phase

**Fig 4:**
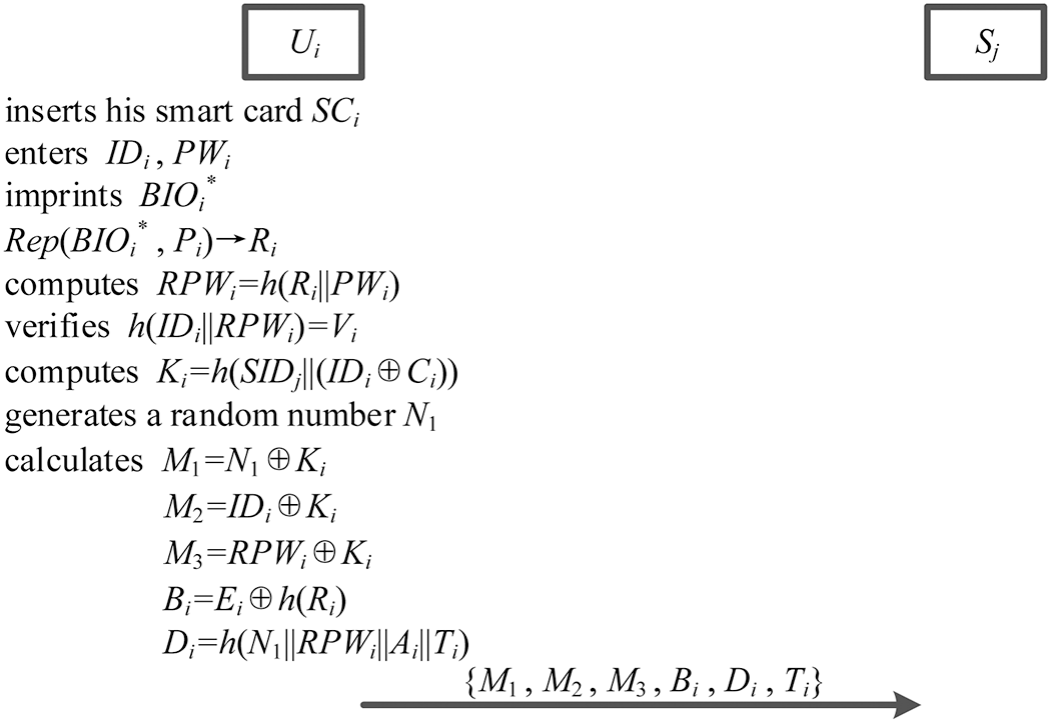
The login phase.

In the login phase, smart card *SC*_*i*_ is able to find the errors immediately by applying user *U*_*i*_’s identity, password, and biometric information. Specifically, login phase is shown in the Fig 4 and details are described as follows.

1. User *U*_*i*_ inserts his smart card *SC*_*i*_ into a smart card reader, enters his identity *ID*_*i*_ and password *PW*_*i*_, and imprints his biometrics $BIO_{i}^{*}$ at a sensor. And then, sensor sketches user *U*_*i*_’s personal biometric information $BIO_{i}^{*}$ and recovers *R*_*i*_ from $Rep\left(BIO_{i}^{*},P_{i}\right)\to R_{i}$ with the assistance of auxiliary binary string *P*_*i*_.

2. Smart card *SC*_*i*_ computes *RPW*_*i*_ = *h*(*R*_*i*_||*PW*_*i*_) and verifies whether *h*(*ID*_*i*_||*RPW*_*i*_) = *V*_*i*_ is valid. If it is valid, smart card *SC*_*i*_ further computes *K*_*i*_ = *h*(*SID*_*j*_||(*ID*_*i*_ ⊕ *C*_*i*_)).

3. Smart card *SC*_*i*_ generates a random number *N*_1_, and calculates *M*_1_ = *N*_1_ ⊕ *K*_*i*_, *M*_2_ = *ID*_*i*_ ⊕ *K*_*i*_, *M*_3_ = *RPW*_*i*_ ⊕ *K*_*i*_, *B*_*i*_ = *E*_*i*_ ⊕ *h*(*R*_*i*_) and *D*_*i*_ = *h*(*N*_1_||*RPW*_*i*_||*A*_*i*_||*T*_*i*_), in which *T*_*i*_ is an additional timestamp.

4. Smart card *SC*_*i*_ submits his login request message {*M*_1_, *M*_2_, *M*_3_, *B*_*i*_, *D*_*i*_, *T*_*i*_} to server *S*_*j*_ over an open channel.

### Authentication phase

**Fig 5:**
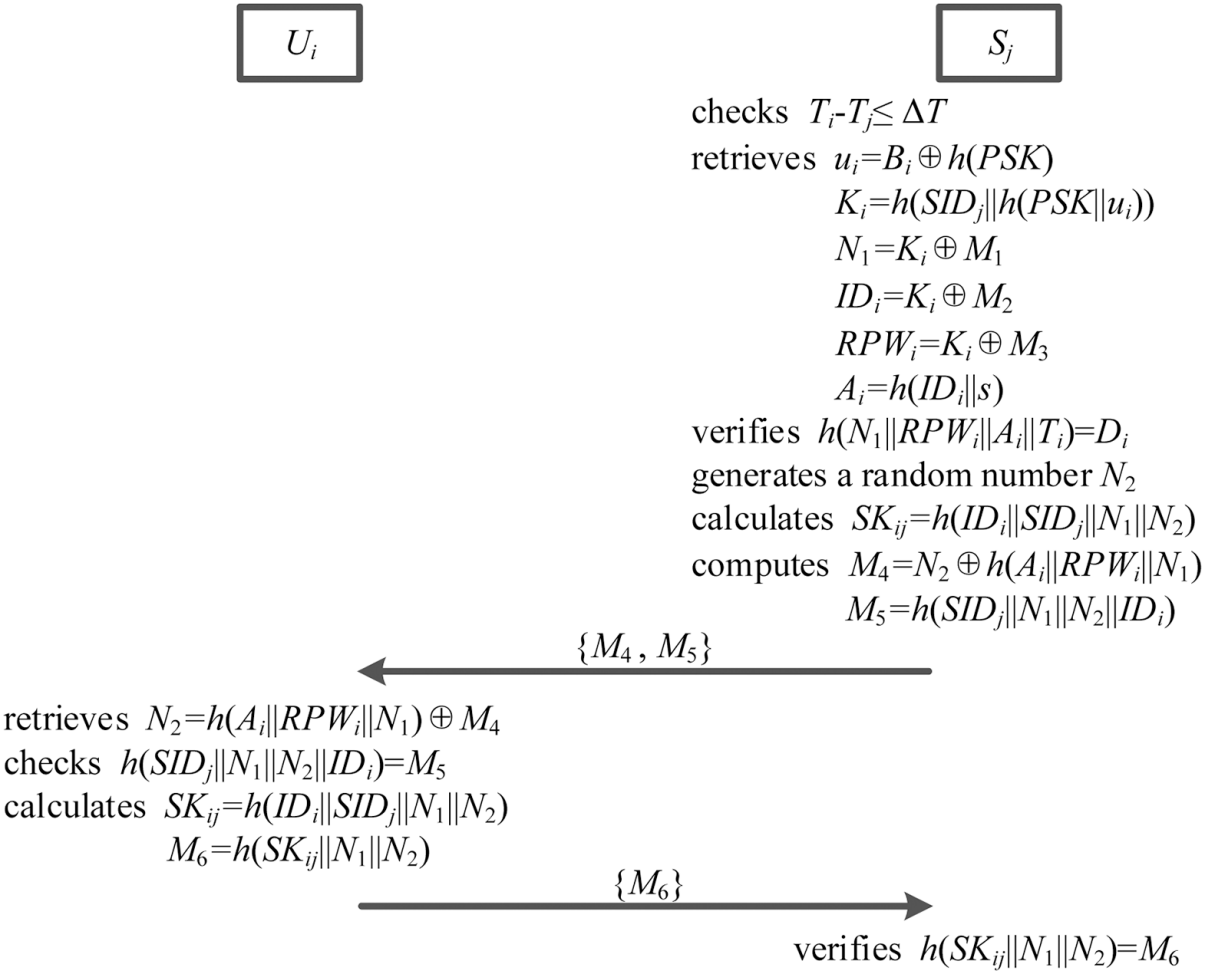
The authentication phase.

During the authentication phase, server *S*_*j*_ has an ability to confirm the destination and freshness of login request message. More details, authentication phase is illustrated in the Fig 5 and explained as below.

1. After receiving user *U*_*i*_’s login request message, server *S*_*j*_ checks whether *T*_*i*_ − *T*_*j*_ ≤ Δ*T* holds, in which Δ*T* is a suitable time interval and *T*_*j*_ is the time when server *S*_*j*_ receives user *U*_*i*_’s login request message. If it holds, server *S*_*j*_ continues to perform the following steps. Otherwise, this login request is rejected by server *S*_*j*_.

2. Server *S*_*j*_ retrieves *u*_*i*_ = *B*_*i*_ ⊕ *h*(*PSK*), *K*_*i*_ = *h*(*SID*_*j*_||*h*(*PSK*||*u*_*i*_)), *N*_1_ = *K*_*i*_ ⊕ *M*_1_, *ID*_*i*_ = *K*_*i*_ ⊕ *M*_2_, *RPW*_*i*_ = *K*_*i*_ ⊕ *M*_3_ and *A*_*i*_ = *h*(*ID*_*i*_||*s*) to verify whether *h*(*N*_1_||*RPW*_*i*_||*A*_*i*_||*T*_*i*_) = *D*_*i*_ is valid.

3. If this verification is valid, server *S*_*j*_ generates another random number *N*_2_, and calculates their session secret key *SK*_*ij*_ = *h*(*ID*_*i*_||*SID*_*j*_||*N*_1_||*N*_2_) between user *U*_*i*_ and server *S*_*j*_.

4. Server *S*_*j*_ computes *M*_4_ = *N*_2_ ⊕ *h*(*A*_*i*_||*RPW*_*i*_||*N*_1_) and *M*_5_ = *h*(*SID*_*j*_||*N*_1_||*N*_2_||*ID*_*i*_), and issues his authentication request message {*M*_4_, *M*_5_} to user *U*_*i*_ through an open channel.

5. When obtaining server *S*_*j*_’s authentication request message, smart card *SC*_*i*_ retrieves *N*_2_ = *h*(*A*_*i*_||*RPW*_*i*_||*N*_1_) ⊕ *M*_4_ and checks whether *h*(*SID*_*j*_||*N*_1_||*N*_2_||*ID*_*i*_) is consistent with *M*_5_. If they are consistent, smart card *SC*_*i*_ calculates *SK*_*ij*_ = *h*(*ID*_*i*_||*SID*_*j*_||*N*_1_||*N*_2_) and *M*_6_ = *h*(*SK*_*ij*_||*N*_1_||*N*_2_). And then smart card *SC*_*i*_ delivers his authentication reply {*M*_6_} to server *S*_*j*_ over a public channel.

6. Server *S*_*j*_ further verifies whether *h*(*SK*_*ij*_||*N*_1_||*N*_2_) = *M*_6_ is valid. If it is valid, server *S*_*j*_ adopts this session key *SK*_*ij*_ to communicate with user *U*_*i*_ in the following communication. Otherwise, authentication will be rejected by *S*_*j*_.

### Password change phase

In the password change phase, user *U*_*i*_ is able to update his password without any help from server *S*_*j*_ or registration center *RC*. More specifically, password change phase includes these following steps.

1. User *U*_*i*_ inputs his identity *ID*_*i*_ and password *PW*_*i*_, and imprints his biometrics $BIO_{i}^{*}$ at a sensor. And then, sensor sketches user *U*_*i*_’s personal biometric information $BIO_{i}^{*}$ and recovers *R*_*i*_ from $Rep\left(BIO_{i}^{*},P_{i}\right)\to R_{i}$ with the assistance of auxiliary binary string *P*_*i*_.

2. Smart card *SC*_*i*_ computes *RPW*_*i*_ = *h*(*R*_*i*_||*PW*_*i*_) and verifies whether *h*(*ID*_*i*_||*RPW*_*i*_) = *V*_*i*_ is valid. If this verification holds, smart card *SC*_*i*_ asks user *U*_*i*_ for a new password. Otherwise, smart card *SC*_*i*_ terminates the password change phase immediately.

3. User *U*_*i*_ enters his new password $PW_{i}^{new}$, and smart card *SC*_*i*_ further calculates $RPW_{i}^{new}=h(R_{i}||PW_{i}^{new})$ and $V_{i}^{new}=h(ID_{i}||RPW_{i}^{new})$.

4. Smart card *SC*_*i*_ replaces *V*_*i*_ with $V_{i}^{new}$ without any help from server *S*_*j*_ or registration center *RC* in the memory.

### User revocation/re-registration phase

If his smart card *SC*_*i*_ is stolen or lost, user revocation/re-registration helps user *U*_*i*_ revoke his privilege or re-register which makes our scheme more robust in the functionality.

1. When user *U*_*i*_ wants to revoke his privilege, he issues his revocation request message, smart card *SC*_*i*_ and verification message {*RPW*_*i*_} to registration center *RC* through a secure channel. Registration center *RC* checks whether user *U*_*i*_ is valid. If user *U*_*i*_ is valid, registration center *RC* further sets 〈*ID*_*i*_, *N*_*i*_ = 0〉 to modify the corresponding entry.

2. Similarly, after obtaining a re-registration request message over a secure channel, registration center *RC* performs these steps mentioned in the subsection 5.2 and helps user *U*_*i*_ re-register by replacing 〈*ID*_*i*_, *N*_*i*_ = *N*_*i*_ + 1〉 with 〈*ID*_*i*_, *N*_*i*_〉.

## Analysis of the proposed scheme

In a multi-server architecture, there are three important requirements for an authentication and key agreement protocol, namely, security, functionality and efficiency. In this section, discussions are performed and results show that our scheme satisfies these requirements mentioned above. Furthermore we compare the proposed protocol with others in respect of security, functionality and efficiency, respectively.

### Informal security analysis

Before the formal security analysis, we analyze the resistance of our scheme against these following attacks by informal security analysis. Remark that adversary *E* has an ability assumed in the threat assumptions to execute these attacks described as follows.

#### Resistance to replay attack

The proposed scheme applies the timestamp and random nonce to endure the replay attack. Though adversary *E* eavesdrops user *U*_*i*_’s previous login request message {*M*_1_, *M*_2_, *M*_3_, *B*_*i*_, *D*_*i*_, *T*_*i*_} and issues it to server *S*_*j*_ as always, server *S*_*j*_ checks the legality of this message by verifying the timeliness of timestamp *T*_*i*_ and correctness of random nonce *N*_1_ as below.

$$\begin{array}{c} D_{i}=h(N_{1}||RPW_{i}\left|\right|A_{i}\left|\right|T_{i}), \end{array}$$

in which both timestamp *T*_*i*_ and random nonce *N*_1_ are different for each session. Thus adversary *E* is rejected by server *S*_*j*_. Therefore our protocol prevents the replay attack.

#### Resistance to Denial-of-Service attack

Adversary *E* tries to diminish or eliminate server *S*_*j*_’s capability by eavesdropping and repeatedly sending user *U*_*i*_’s previous login request message. However, server *S*_*j*_ verifies the freshness of timestamp *T*_*i*_ and checks whether *D*_*i*_ = *h*(*N*_1_||*RPW*_*i*_||*A*_*i*_||*T*_*i*_) holds. So server *S*_*j*_ treats adversary *E* as a malicious hacker and terminates this session. Furthermore the presented scheme introduces a biometrics-based fuzzy extractor to meet the applicability of biometric information. Consequently, our protocol resists the Denial-of-Service attack.

#### Resistance to password guessing attack

With the assistance of power consumption, adversary *E* applies the side-channel attacks, such as SPA or DPA, to extract the sensitive datas *A*_*i*_, *C*_*i*_, *E*_*i*_, *V*_*i*_ and *P*_*i*_ from user *U*_*i*_’s smart card *SC*_*i*_. But he is unable to verify whether user *U*_*i*_’s password *PW*_*i*_ is correct in the on-line or off-line environment without biometric information *BIO*_*i*_, pre shared key *PSK*, master secret key *s* and random nonce *N*_1_. Specifically unpredictable binary string *R*_*i*_ which possesses a high entropy protects user *U*_*i*_’s password *PW*_*i*_ in the proposed scheme. In conclusion, our protocol is secure against the password guessing attack.

#### Resistance to smart card attack

Without the password *PW*_*i*_ or biometric information *BIO*_*i*_, adversary *E* launches the smart card attack in order to collect some sensitive datas stored in the smart card *SC*_*i*_ and achieve server *S*_*j*_’s authentication. In the presented scheme, adversary *E* is able to acquire user *U*_*i*_’s sensitive datas *A*_*i*_, *C*_*i*_, *E*_*i*_, *V*_*i*_ and *P*_*i*_ which are saved in the smart card *SC*_*i*_ by SPA or DPA. Also a session key *SK*_*ij*_ between user *U*_*i*_ and server *S*_*j*_ is calculated as follows.

$$\begin{array}{c} K_{i}=h(SID_{j}\left|\right|\left(ID_{i}⊕C_{i}\right)), \end{array}$$

$$\begin{array}{c} N_{1}=K_{i}⊕M_{1}, \end{array}$$

$$\begin{array}{c} N_{2}=h(A_{i}||RPW_{i}\left|\right|N_{1})⊕M_{4}, \end{array}$$

$$\begin{array}{c} SK_{ij}=h(ID_{i}||SID_{j}\left|\right|N_{1}\left|\right|N_{2}). \end{array}$$

It is feasible for adversary *E* to obtain *M*_1_ and *M*_4_ through a public channel. However, it is pretty difficult for him to retrieve the random nonces *N*_1_ or *N*_2_. As a result, our protocol withstands the smart card attack.

#### Resistance to user impersonation attack

Under the user impersonation attack, adversary *E* who is an outside hacker tries to impersonate user *U*_*i*_ without the password *PW*_*i*_ or biometric information *BIO*_*i*_. In the proposed scheme, adversary *E* is unable to acquire *h*(*PSK*) even if he eavesdrops user *U*_*i*_’s previous login request message {*M*_1_, *M*_2_, *M*_3_, *B*_*i*_, *D*_*i*_, *T*_*i*_} and extracts user *U*_*i*_’s sensitive datas from smart card *SC*_*i*_ by SPA or DPA. Thus, adversary *E* cannot retrieve the random numbers *N*_1_, *N*_2_ or session key *SK*_*ij*_. Therefore, our protocol is secure against the user impersonation attack.

#### Resistance to privileged insider attack

Adversary *E* who is a malicious insider and has a privilege to access an authorized system attempts to impersonate user *U*_*i*_. In order to achieve this goal, adversary *E* collects user *U*_*i*_’s registration request message {*ID*_*i*_, *RPW*_*i*_} and steals his smart card *SC*_*i*_. However, it is impossible to obtain *h*(*PSK*) and *B*_*i*_ for adversary *E*. Even if sensitive datas *A*_*i*_, *C*_*i*_, *E*_*i*_, *V*_*i*_ and *P*_*i*_ are extracted from user *U*_*i*_’s smart card *SC*_*i*_, adversary *E* is unable to deliver a correct login request message {*M*_1_, *M*_2_, *M*_3_, *B*_*i*_, *D*_*i*_, *T*_*i*_}. Furthermore, he cannot retrieve the password *PW*_*i*_ or biometric information *BIO*_*i*_. In conclusion, our protocol resists the privileged insider attack.

#### Resistance to server spoofing attack

Under the assumption that adversary *E* who is a malicious insider but isn’t another server *S*_*k*_ is able to steal user *U*_*i*_’s smart card *SC*_*i*_ and eavesdrop his registration request message {*ID*_*i*_, *RPW*_*i*_}. Adversary *E* tries to masquerade as server *S*_*j*_ to spoof user *U*_*i*_ by collecting the sensitive datas *A*_*i*_, *C*_*i*_, *E*_*i*_, *V*_*i*_ and *P*_*i*_. But it is hard to retrieve *h*(*PSK*) so that adversary *E* is unable to be authenticated by user *U*_*i*_ successfully. He cannot acquire the random number *N*_1_ and valid authentication request message {*M*_4_, *M*_5_}. Thus adversary *E*’s attempt fails. Consequently, our protocol prevents the server spoofing attack.

#### Resistance to modification attack

Though adversary *E* attempts to modify some intercepted messages for further authentication, the proposed protocol is able to check whether the received messages are valid with the assistance of collision-resistant hash function. And adversary *E* does not have a capability to retrieve *N*_1_, *N*_2_ or *h*(*PSK*) from any intercepted message. Thus he cannot generate a legitimate authentication message. As a result, our protocol is secure against the modification attack.

#### Resistance to stolen-verifier attack

In the proposed protocol, both server *S*_*j*_ and registration center *RC* possess no information about user *U*_*i*_’s password or biometrics. Concretely, there is no password-verifier or biometrics-verifier in the database of server *S*_*j*_ and registration center *RC*. Thus, adversary *E* cannot launch the stolen-verifier attack even if he has an authority to access the database. Consequently, our protocol withstands the stolen-verifier attack.

#### Possession of anonymity

During the login phase of the proposed scheme, user *U*_*i*_ calculates his dynamic identity *M*_2_ = *ID*_*i*_ ⊕ *K*_*i*_, in which *K*_*i*_ cannot be retrieved by adversary *E* from any request or reply message. Thus, adversary *E* has no ability to acquire user *U*_*i*_’s identity *ID*_*i*_. However, upon receiving user *U*_*i*_’s login request message, authorized server *S*_*j*_ calculates *u*_*i*_ = *B*_*i*_ ⊕ *h*(*PSK*) and further computes *K*_*i*_ = *h*(*SID*_*j*_||*h*(*PSK*||*u*_*i*_)) so that user *U*_*i*_ achieves server *S*_*j*_’s authentication anonymously. In other words, user *U*_*i*_’s real identity *ID*_*i*_ is not disclosed by any unauthorized participant. Therefore our protocol provides the anonymity.

#### Possession of perfect forward secrecy

Perfect forward secrecy protects the session keys even if long-term key is retrieved. Specifically, session key *SK*_*ij*_ in the proposed scheme is generated as follows.

$$\begin{array}{c} K_{i}=h(SID_{j}\left||h(PSK|\right|u_{i})), \end{array}$$

$$\begin{array}{c} N_{1}=K_{i}⊕M_{1}, \end{array}$$

$$\begin{array}{c} ID_{i}=K_{i}⊕M_{2}, \end{array}$$

$$\begin{array}{c} N_{2}=h(A_{i}||RPW_{i}\left|\right|N_{1})⊕M_{4}, \end{array}$$

$$\begin{array}{c} SK_{ij}=h(ID_{i}||SID_{j}\left|\right|N_{1}\left|\right|N_{2}). \end{array}$$

Though the long-term key *h*(*PSK*) is calculated by adversary *E*, it is impossible to compute some sensitive datas, such as *RPW*_*i*_, *K*_*i*_ and *PSK*. Thus adversary *E* is unable to obtain the random numbers *N*_1_ or *N*_2_. Also it is hard for adversary *E* to retrieve the session key *SK*_*ij*_ between user *U*_*i*_ and server *S*_*j*_. Therefore, our protocol provides the perfect forward secrecy.

### Formal security analysis

During this subsection, we provide a formal security analysis and demonstrate that the proposed scheme is secure. In order to achieve this purpose, we define the oracle *Reveal* as below. It unconditionally retrieves the original input *x* from the collision-resistant hash function *y* = *h*(*x*). More details relating to this formal security analysis are shown in the following theorem.

**Theorem.** Suppose that the collision-resistant hash function *h*(⋅) operates closely like the oracle *Reveal*, our protocol is provably secure to protect the sensitive datas which include registration center *RC*’s master secret key *s*, pre shared key *PSK* between registration center *RC* and server *S*_*j*_, user *U*_*i*_’s identity *ID*_*i*_ and password *PW*_*i*_.

**Proof.** With the assistance of the oracle *Reveal*, we make an assumption that adversary *E* has a capacity to retrieve registration center *RC*’s master secret key *s*, pre shared key *PSK* between registration center *RC* and server *S*_*j*_, user *U*_*i*_’s identity *ID*_*i*_ and password *PW*_*i*_. Adversary *E* executes the following experimental algorithm $EXP_{E,AKAS}^{HASH}$, in which *AKAS* means the presented scheme. More details about the Algorithm $EXP_{E,AKAS}^{HASH}$ are explained in the Table 3

**Table 3:** Algorithm $EXP_{E,AKAS}^{HASH}$.

01. Eavesdrop user *U*_*i*_’s login request message {*M*_1_, *M*_2_, *M*_3_, *B*_*i*_, *D*_*i*_, *T*_*i*_} in the login phase,
in which *B*_*i*_ = *E*_*i*_ ⊕ *h*(*R*_*i*_), *D*_*i*_ = *h*(*N*_1_||*RPW*_*i*_||*A*_*i*_||*T*_*i*_), *M*_1_ = *N*_1_ ⊕ *K*_*i*_, *M*_2_ = *ID*_*i*_ ⊕ *K*_*i*_ and *M*_3_ = *RPW*_*i*_ ⊕ *K*_*i*_.
02. Apply this oracle *Reveal* to extract some values $N_{1}^{I}$, $RPW_{i}^{I}$, $A_{i}^{I}$ and $T_{i}^{I}$ from $Reveal\left(D_{i}\right)\to (N_{1}^{I}||RPW_{i}^{I}\left|\right|A_{i}^{I}\left|\right|T_{i}^{I})$.
03. Eavesdrop server *S*_*j*_’s authentication request message {*M*_4_, *M*_5_} during the authentication phase,
in which *M*_4_ = *N*_2_ ⊕ *h*(*A*_*i*_||*RPW*_*i*_||*N*_1_) and *M*_5_ = *h*(*SID*_*j*_||*N*_1_||*N*_2_||*ID*_*i*_).
04. Apply this oracle *Reveal* to extract some values $SID_{j}^{II}$, $N_{1}^{II}$, $N_{2}^{II}$ and $ID_{i}^{II}$ from $Reveal\left(M_{5}\right)\to (SID_{j}^{II}\left|\right|N_{1}^{II}\left|\right|N_{2}^{II}||ID_{i}^{II})$.
05. **if** ($N_{1}^{I}=N_{1}^{II}$) **then**
06. Apply this oracle *Reveal* to extract some values $R_{i}^{I}$ and $PW_{i}^{I}$ from $Reveal\left(RPW_{i}^{I}\right)\to (R_{i}^{I}||PW_{i}^{I})$.
07. Further apply this oracle *Reveal* to extract some values $ID_{i}^{I}$ and *s*^*I*^ from $Reveal\left(A_{i}^{I}\right)\to (ID_{i}^{I}\left|\right|s^{I})$.
08. Calculate $K_{i}^{I}=M_{1}⊕N_{1}^{I}$.
09. Further calculate $K_{i}^{II}=M_{1}⊕N_{1}^{II}$.
10. **if** ($K_{i}^{I}=K_{i}^{II}$) **then**
11. Apply this oracle *Reveal* to extract some values $SID_{j}^{I}$ and *h*(*PSK*||*u*_*i*_)^*I*^ from $Reveal\left(K_{i}^{I}\right)\to (SID_{j}^{I}||h{\left(PSK||u_{i}\right)}^{I})$.
12. Further apply this oracle *Reveal* to extract some values *PSK*^*I*^ and $u_{i}^{I}$ from $Reveal\left(h{\left(PSK||u_{i}\right)}^{I}\right)\to (PSK^{I}\left|\right|u_{i}^{I})$.
13. Calculate $N_{2}^{I}=h(A_{i}^{I}||RPW_{i}^{I}\left|\right|N_{1}^{I})⊕M_{4}$.
14. **if** ($N_{2}^{I}=N_{2}^{II}$) **then**
15. Accept *s*^*I*^, *PSK*^*I*^, $ID_{i}^{I}$ and $PW_{i}^{I}$ as registration center *RC*’s master secret key *s*,
pre shared key *PSK* between registration center *RC* and server *S*_*j*_, user *U*_*i*_’s identity *ID*_*i*_ and password *PW*_*i*_, respectively.
16. **return** 1 (Success)
17. **else**
18. **return** 0 (Failure)
19. **end if**
20. **else**
21. **return** 0 (Failure)
22. **end if**
23. **else**
24. **return** 0 (Failure)
25. **end if**

Furthermore, we define a success probability about $EXP_{E,AKAS}^{HASH}$ as $Success=|P(EXP_{E,AKAS}^{HASH}=1)-1|$. Thus advantage function of algorithm $EXP_{E,AKAS}^{HASH}$ is *Adv*(*et*, *q*_*Reveal*_) = max_*E*_{*Success*}, namely, maximum for adversary *E* relies on the execution time *et* and query counts *q*_*Reveal*_ which are made to this oracle *Reveal*. If *Adv*(*et*, *q*_*Reveal*_) ≤ *ε*, our protocol is secure against adversary *E* for any sufficiently small *ε* > 0. It enables adversary *E* to win this game if it is possible to retrieve the original input *x* from the collision-resistant hash function *y* = *h*(*x*). However, it is a computationally infeasible problem for retrieving the original input *x*. Therefore, for any sufficiently small *ε* > 0, max_*E*_{*Success*} = *Adv*(*et*, *q*_*Reveal*_) ≤ *ε*. As a result, our protocol is provably secure to protect registration center *RC*’s master secret key *s*, pre shared key *PSK* between registration center *RC* and server *S*_*j*_, user *U*_*i*_’s identity *ID*_*i*_ and password *PW*_*i*_.

### Security analysis with BAN logic

As an important verification tool, Burrows-Abadi-Needham (BAN) logic has a set of rules [66]. In the security analysis, BAN logic is used for defining and analyzing the information exchange schemes, especially authentication and key agreement protocols. Particularly, BAN logic is able to verify whether exchanged information is trustworthy [67]. During this subsection, we apply BAN logic to prove that session key *SK*_*ij*_ between server *S*_*j*_ and user *U*_*i*_ is correctly generated during the authentication phase of our protocol. For convenience, symbols and corresponding notions about BAN logic are respectively shown in Table 4.

**Table 4:** Symbols and corresponding notions in the BAN logic.

Symbol	Notion
*A*| ≡ *X*	Principal *A* believes the truth of statement *X*.
$A\overset{K}{⟷}B$	Principal *A* and principal *B* share session key *K*.
*A* ⇒ *X*	Principal *A* has a jurisdiction over the truth of statement *X*.
#*X*	Statement *X* is fresh.
*A* ⊲ *X*	Principal *A* sees the statement *X*.
*A*|∼*X*	Principal *A* once said the statement *X*.
{*X*, *Y*}_*K*_	Statement *X* and statement *Y* are encrypted by session key *K*.
(*X*, *Y*)_*K*_	Statement *X* and statement *Y* are hashed by session key *K*.
<*X*>_*K*_	Statement *X* is XORed by session key *K*.

#### The BAN logical postulates

1. The message-meaning rule, namely, $\frac{A|\equiv A\overset{K}{⟷}B,A⊴{\left\{X\right\}}_{K}}{A|\equiv B|∼X}$. Particularly, if principal *A* believes that principal *A* and principal *B* share session key *K*, and principal *A* sees that statement *X* is encrypted by session key *K*, then principal *A* believes that principal *B* once said the statement *X*.

2. The nonce-verification rule, namely, $\frac{A|\equiv \#X,A|\equiv B|∼X}{A|\equiv B|\equiv X}$. Specifically, if principal *A* believes that statement *X* is fresh and principal *B* once said the statement *X*, then principal *A* believes that principal *B* believes the truth of statement *X*.

3. The belief rule, namely, $\frac{A|\equiv X,A|\equiv Y}{A|\equiv (X,Y)}$. In particular, if principal *A* believes the truth of statement *X* and statement *Y*, then principal *A* believes the truth of (*X*, *Y*).

4. The freshness-conjuncatenation rule, namely, $\frac{A|\equiv \#X}{A|\equiv \#(X,Y)}$. Concretely, if principal *A* believes that statement *X* is fresh, then principal *A* believes that (*X*, *Y*) is fresh.

5. The jurisdiction rule, namely, $\frac{A|\equiv B\Rightarrow X,A|\equiv B|\equiv X}{A|\equiv X}$. Especially, if principal *A* believes that principal *B* has a jurisdiction over the truth of statement *X* and principal *B* believes the truth of statement *X*, then principal *A* believes the truth of statement *X*.

#### The idealized scheme

*U*_*i*_: <*N*_1_, *ID*_*i*_, *RPW*_*i*_>_*K*_*i*__, (*N*_1_, *A*_*i*_, *T*_*i*_)_*RPW*_*i*__ and ${\left(U_{i}\overset{SK_{ij}}{⟷}S_{j},N_{2}\right)}_{N_{1}}$.

*S*_*j*_: <*A*_*i*_, *RPW*_*i*_, *N*_1_>_*N*_2__ and (*ID*_*i*_, *N*_1_, *N*_2_)_*SID*_*j*__.

#### The establishment of security goals

*g*1. $U_{i}\left|\equiv S_{j}\right|\equiv U_{i}\overset{SK_{ij}}{⟷}S_{j}$

*g*2. $U_{i}|\equiv U_{i}\overset{SK_{ij}}{⟷}S_{j}$

*g*3. $S_{j}\left|\equiv U_{i}\right|\equiv U_{i}\overset{SK_{ij}}{⟷}S_{j}$

*g*4. $S_{j}|\equiv U_{i}\overset{SK_{ij}}{⟷}S_{j}$

#### The initiative premises

*p*1. *U*_*i*_| ≡ #*N*_1_

*p*2. *U*_*i*_| ≡ *S*_*j*_ ⇒ #*N*_2_

*p*3. *S*_*j*_| ≡ #*N*_1_

*p*4. *S*_*j*_| ≡ #*N*_2_

*p*5. $S_{j}|\equiv U_{i}\overset{K_{i}}{⟷}S_{j}$

*p*6. $U_{i}|\equiv U_{i}\overset{SID_{j}}{⟷}S_{j}$

*p*7. *U*_*i*_| ≡ *ID*_*i*_

*p*8. *S*_*j*_| ≡ *U*_*i*_ ⇒ *RPW*_*i*_

*p*9. *S*_*j*_| ≡ *U*_*i*_ ⇒ *ID*_*i*_

*p*10. $S_{j}|\equiv U_{i}\overset{N_{1}}{⟷}S_{j}$

*p*11. $S_{j}|\equiv U_{i}\Rightarrow U_{i}\overset{SK_{ij}}{⟷}S_{j}$

*p*12. $U_{i}|\equiv S_{j}\Rightarrow U_{i}\overset{SK_{ij}}{⟷}S_{j}$

#### The security analysis

*a*1. Because of *p*5 and *S*_*j*_ ⊲ <*N*_1_, *ID*_*i*_, *RPW*_*i*_>_*K*_*i*__, we execute the message-meaning rule to obtain *S*_*j*_| ≡ *U*_*i*_| ∼ (*N*_1_, *ID*_*i*_, *RPW*_*i*_).

*a*2. Since *p*3 and *a*1, we adopt both freshness-conjuncatenation rule and nonce-verification rule to acquire *S*_*j*_| ≡ *U*_*i*_| ≡ (*N*_1_, *ID*_*i*_, *RPW*_*i*_).

*a*3. Because of *p*10 and $S_{j}⊴{\left(U_{i}\overset{SK_{ij}}{⟷}S_{j},N_{2}\right)}_{N_{1}}$, we use the message-meaning rule to derive $S_{j}\left|\equiv U_{i}\right|∼\left(U_{i}\overset{SK_{ij}}{⟷}S_{j},N_{2}\right)$.

*a*4. Since *p*4 and *a*3, we apply both freshness-conjuncatenation rule and nonce-verification rule to get $S_{j}\left|\equiv U_{i}\right|\equiv \left(U_{i}\overset{SK_{ij}}{⟷}S_{j},N_{2}\right)$.

*g*3. Because of *a*4, we execute the belief rule to obtain $S_{j}\left|\equiv U_{i}\right|\equiv U_{i}\overset{SK_{ij}}{⟷}S_{j}$.

*g*4. Since *p*11 and *g*3, we adopt the jurisdiction rule to acquire $S_{j}|\equiv U_{i}\overset{SK_{ij}}{⟷}S_{j}$.

*a*5. Because of *p*6 and *U*_*i*_ ⊲ (*ID*_*i*_, *N*_1_, *N*_2_)_*SID*_*j*__, we use the message-meaning rule to derive *U*_*i*_| ≡ *S*_*j*_|∼(*ID*_*i*_, *N*_1_, *N*_2_).

*a*6. Since *p*2 and *a*5, we apply both freshness-conjuncatenation rule and nonce-verification rule to get *U*_*i*_| ≡ *S*_*j*_| ≡ (*ID*_*i*_, *N*_1_, *N*_2_).

*a*7. Because of *a*6, we execute the belief rule to obtain *U*_*i*_| ≡ *S*_*j*_| ≡ *N*_2_.

*a*8. Since *p*2 and *a*7, we adopt the jurisdiction rule to acquire *U*_*i*_| ≡ *N*_2_.

*a*9. Because of *p*8, *p*9 and *a*2, we execute both belief rule and jurisdiction rule to obtain *S*_*j*_| ≡ *ID*_*i*_.

*g*1. Since *p*1, *p*3, *p*4, *p*6, *p*7, *a*8, *a*9 and *SK*_*ij*_ = *h*(*ID*_*i*_||*SID*_*j*_||*N*_1_||*N*_2_), we adopt both freshness-conjuncatenation rule and nonce-verification rule to acquire $U_{i}\left|\equiv S_{j}\right|\equiv U_{i}\overset{SK_{ij}}{⟷}S_{j}$.

*g*2. Because of *g*1 and *p*12, we use the jurisdiction rule to derive $U_{i}|\equiv U_{i}\overset{SK_{ij}}{⟷}S_{j}$.

Above all, results mentioned above demonstrate that our protocol enables to generate the shared session key *SK*_*ij*_ correctly between server *S*_*j*_ and user *U*_*i*_.

### Functionality analysis

It is necessary to meet the functionality requirements which include mutual authentication, session key agreement, user revocation/re-registration and biometric information protection. In this section, we demonstrate that our protocol provides all functionality mentioned above. More details relating to functionality analysis are shown as below.

#### Mutual authentication

In the presented scheme, both user *U*_*i*_ and server *S*_*j*_ authenticate each other by taking advantage of some sensitive datas, for example *N*_1_, *N*_2_, *K*_*i*_, *T*_*i*_ and *SK*_*ij*_. In particular, server *S*_*j*_ checks whether *h*(*N*_1_||*RPW*_*i*_||*A*_*i*_||*T*_*i*_) = *D*_*i*_ and *h*(*SK*_*ij*_||*N*_1_||*N*_2_) = *M*_6_ are valid. Similarly, user *U*_*i*_ verifies whether *h*(*SID*_*j*_||*N*_1_||*N*_2_||*ID*_*i*_) is consistent with *M*_5_. As a result, our protocol achieves the mutual authentication.

#### Session key agreement

During the authentication phase, session key *SK*_*ij*_ = *h*(*ID*_*i*_||*SID*_*j*_||*N*_1_||*N*_2_) between server *S*_*j*_ and user *U*_*i*_ is established to protect the subsequent communications. Especially, both *N*_1_ and *N*_2_ change in every authentication phase so that session key *SK*_*ij*_ is different during each session. Furthermore it is hard to retrieve their session key *SK*_*ij*_ for adversary *E*. In conclusion, our protocol possesses the session key agreement.

#### User revocation/re-registration

It is necessary for user *U*_*i*_ to revoke or re-register his privilege. In the presented scheme, registration center *RC* helps user *U*_*i*_ achieve the user revocation/re-registration by modifying the entry 〈*ID*_*i*_, *N*_*i*_〉 when obtaining user *U*_*i*_’s revocation or re-registration request message via a secure channel. Above all, our protocol achieves the user revocation/re-registration.

#### Biometric information protection

In some conventional schemes, user *U*_*i*_’s biometric information *BIO*_*i*_ is directly stored in his smart card *SC*_*i*_ without appropriate protection. Thus adversary *E* is able to extract user *U*_*i*_’s biometrics *BIO*_*i*_ from a lost or stolen smart card *SC*_*i*_ through side channel attacks. In order to solve this problem, we apply a high error-tolerant mechanism to save user *U*_*i*_’s biometric information *BIO*_*i*_. Besides, collision-resistant hash function protects the unpredictable binary string *R*_*i*_. So it is impossible for adversary *E* to extract user *U*_*i*_’s biometric information *BIO*_*i*_. In conclusion, our protocol possesses the biometric information protection.

### Efficiency analysis

In this subsection, we estimate the storage requirement, communication overhead and computational cost of the presented scheme. More details about efficiency analysis are shown as below.

#### Storage requirement

For the storage requirement, we apply these messages which are stored in user *U*_*i*_’s smart card *SC*_*i*_ as storage overhead. Particularly, byte length of nonce both *N*_1_ and *N*_2_ is 20, byte length of user *U*_*i*_’s identity *ID*_*i*_ is 20, byte length of timestamp *T*_*i*_ is 2 and byte length of collision-resistant hash function’s output is 20 if we apply the SHA-1. Thus, we are able to calculate the byte length of stored datas in the proposed scheme. As a result, all saved messages {*A*_*i*_, *C*_*i*_, *E*_*i*_, *V*_*i*_, *P*_*i*_} require 20 + 20 + 20 + 20 + 20 = 100 bytes in respect of storage need.

#### Communication overhead

In order to estimate the communication overhead, we consider user *U*_*i*_’s login request message {*M*_1_, *M*_2_, *M*_3_, *B*_*i*_, *D*_*i*_, *T*_*i*_} which is submitted to server *S*_*j*_ in the stage of login. According to assumption described above, length of this message is 20 + 20 + 20 + 20 + 20 + 2 = 102 bytes. Similarly, communication overhead that includes server *S*_*j*_’s authentication request message {*M*_4_, *M*_5_} and user *U*_*i*_’s authentication reply {*M*_6_} is 20 + 20 + 20 = 60 bytes during the authentication phase. Therefore, total communication overhead of our protocol is 102 + 60 = 162 bytes.

#### Computational cost

Considering the computational complexity, we apply the frequency of collision-resistant hash function as computational cost. Besides, it is practicable to ignore the computational complexity of XOR operation which requires very little time. In the environment where CPU is 2.20 GHz and RAM is 2048 MB, it takes 0.0023 ms to execute the collision-resistant hash function on average [55, 68]. In the presented scheme, we execute the collision-resistant hash function four times and thirteen times in the login phase and authentication phase, respectively. Above all, our protocol requires 0.0115 + 0.0299 = 0.0414 ms for computational cost.

### Comparisons with related schemes

During this section, we compare the proposed protocol with other related schemes in terms of security, functionality and efficiency. In particular, our protocol is compared with some multi-server authentication schemes, such as Mishra et al.’s scheme [50], Lin et al.’s scheme [53], Wang et al.’s scheme [61], Chaudhry et al.’s scheme [64], Chaudhry et al.’s scheme [41] and Khan et al.’s scheme [65]. Results ensure that the presented protocol is efficient in these aspects mentioned above.

In particular, Table 5 lists the security comparison between various authentication schemes and ours. For convenience, we define some following notations in the Table 5, where R1 represents the resistance to replay attack, R2 represents the resistance to Denial-of-Service attack, R3 represents the resistance to password guessing attack, R4 represents the resistance to smart card attack, R5 represents the resistance to user impersonation attack, R6 represents the resistance to privileged insider attack, R7 represents the resistance to server spoofing attack, R8 represents the resistance to modification attack, R9 represents the resistance to stolen-verifier attack, R10 represents the possession of anonymity and R11 represents the possession of perfect forward secrecy. Concretely, Mishra et al.’s scheme [50] cannot resist the replay attack, Denial-of-Service attack, smart card attack, user impersonation attack, privileged insider attack and server spoofing attack. Also their scheme is unable to provide the anonymity and perfect forward secrecy. According to the cryptanalysis in Ref. [69], Lin et al.’s scheme [53] is insecure against the user impersonation attack and server spoofing attack. And their scheme fails to possess the anonymity. Wang et al.’s scheme [61] cannot prevent the user impersonation attack, privileged insider attack and server spoofing attack. Also their scheme is unable to achieve the perfect forward secrecy. Due to the cryptanalysis in Ref. [70], Chaudhry et al.’s scheme [64] is insecure against the Denial-of-Service attack and cannot provide the perfect forward secrecy. Consequently, result demonstrates that our protocol achieves all security properties.

**Table 5:** The security comparison.

	Ref. [50]	Ref. [53]	Ref. [61]	Ref. [64]	Ref. [41]	Ref. [65]	Ours
R1	No	Yes	Yes	Yes	Yes	Yes	Yes
R2	No	Yes	Yes	No	Yes	Yes	Yes
R3	Yes	Yes	Yes	Yes	Yes	Yes	Yes
R4	No	Yes	Yes	Yes	Yes	Yes	Yes
R5	No	No	No	Yes	Yes	Yes	Yes
R6	No	Yes	No	Yes	Yes	Yes	Yes
R7	No	No	No	Yes	Yes	Yes	Yes
R8	Yes	Yes	Yes	Yes	Yes	Yes	Yes
R9	Yes	Yes	Yes	Yes	Yes	Yes	Yes
R10	No	No	Yes	Yes	Yes	Yes	Yes
R11	No	Yes	No	No	Yes	Yes	Yes

Besides, Table 6 shows the functionality comparison between some related schemes and ours. Also we further compare our protocol with Reddy et al.’s scheme [69] and Irshad et al.’s scheme [71] which are other improved schemes. In the Table 6, we apply some following notations, where F1 represents the mutual authentication, F2 represents the session key agreement, F3 represents the user revocation/re-registration and F4 represents the biometric information protection. Concretely, Mishra et al.’s scheme [50] cannot provide the user revocation/re-registration. Similarly, Lin et al.’s scheme [53] fails to achieve the user revocation/re-registration. As a result, our protocol provides more functionality properties.

**Table 6:** The functionality comparison.

	Ref. [50]	Ref. [53]	Ref. [61]	Ref. [64]	Ref. [41]	Ref. [65]	Ref. [69]	Ref. [71]	Ours
F1	Yes	Yes	Yes	Yes	Yes	Yes	Yes	Yes	Yes
F2	Yes	Yes	Yes	Yes	Yes	Yes	Yes	Yes	Yes
F3	No	No	Yes	Yes	Yes	Yes	Yes	Yes	Yes
F4	Yes	Yes	Yes	Yes	Yes	Yes	Yes	Yes	Yes

**Fig 6:**
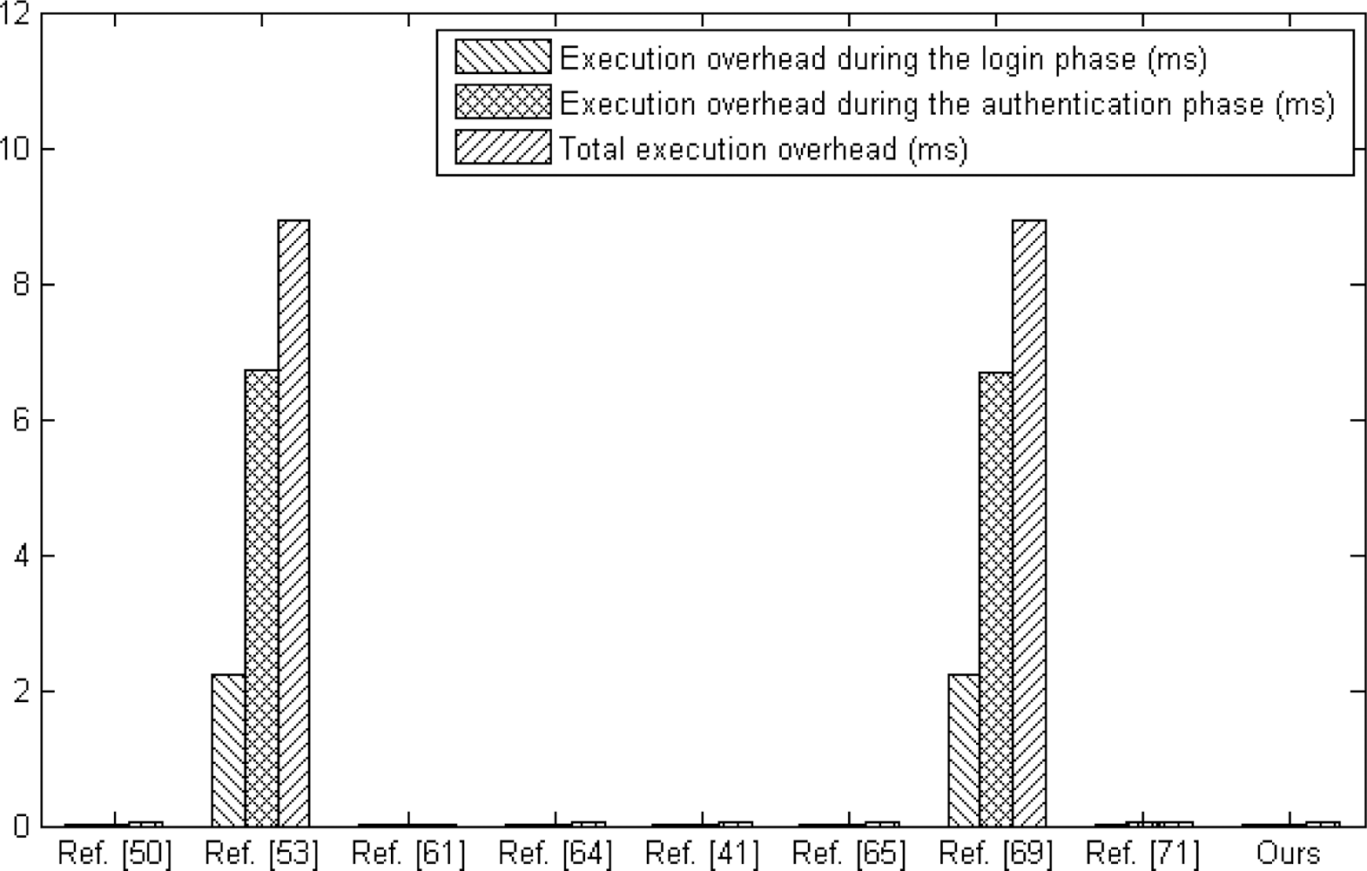
The computation cost comparison.

Specifically, Table 7 and Fig 6 indicate the computational cost comparison between various related schemes and ours involved in both login phase and authentication phase. As a convenience, we define some following notations in the Table 7, where C1 represents the computational cost during the login phase, C2 represents the execution overhead during the login phase, C3 represents the computational cost during the authentication phase, C4 represents the execution overhead during the authentication phase and C5 represents the total execution overhead. Besides, *T*_*h*_ represents the computation time for collision-resistant hash function, *T*_*p*_ represents the computation time for point multiplication based on elliptic curve, *T*_*s*_ represents the computation time for symmetric encryption/decryption and *T*_*c*_ represents the computation time for Chebyshev chaotic map. According to the execution overhead given in [55] and [68], in the environment where CPU is 2.20 GHz and RAM is 2048 MB, it spends about 2.2260 ms, 0.0046 ms and 0.0045 ms to execute the point multiplication based on elliptic curve, symmetric encryption/decryption and Chebyshev chaotic map, respectively. Compared with other schemes, result indicates that our protocol requires the lower computational cost.

**Table 7:** The computational cost comparison.

	Ref. [50]	Ref. [53]	Ref. [61]	Ref. [64]	Ref. [41]	Ref. [65]	Ref. [69]	Ref. [71]	Ours
C1	7*T*_*h*_	3*T*_*h*_ + 1*T*_*p*_ + 2*T*_*s*_	4*T*_*h*_	5*T*_*h*_	4*T*_*h*_ + 1*T*_*s*_	4*T*_*h*_ + 2*T*_*c*_	6*T*_*h*_ + 1*T*_*p*_	9*T*_*h*_	5*T*_*h*_
C2	0.0161 ms	2.2421 ms	0.0092 ms	0.0115 ms	0.0138 ms	0.0182 ms	2.2398 ms	0.0207 ms	0.0115 ms
C3	11*T*_*h*_	5*T*_*h*_ + 3*T*_*p*_ + 3*T*_*s*_	11*T*_*h*_	7*T*_*h*_ + 2*T*_*s*_	8*T*_*h*_ + 1*T*_*s*_	6*T*_*h*_ + 4*T*_*c*_	9*T*_*h*_ + 3*T*_*p*_	12*T*_*h*_ + 2*T*_*s*_	13*T*_*h*_
C4	0.0253 ms	6.7033 ms	0.0253 ms	0.0253 ms	0.0230 ms	0.0318 ms	6.6987 ms	0.0368 ms	0.0299 ms
C5	0.0414 ms	8.9454 ms	0.0345 ms	0.0368 ms	0.0368 ms	0.0500 ms	8.9385 ms	0.0575 ms	0.0414 ms

**Fig 7:**
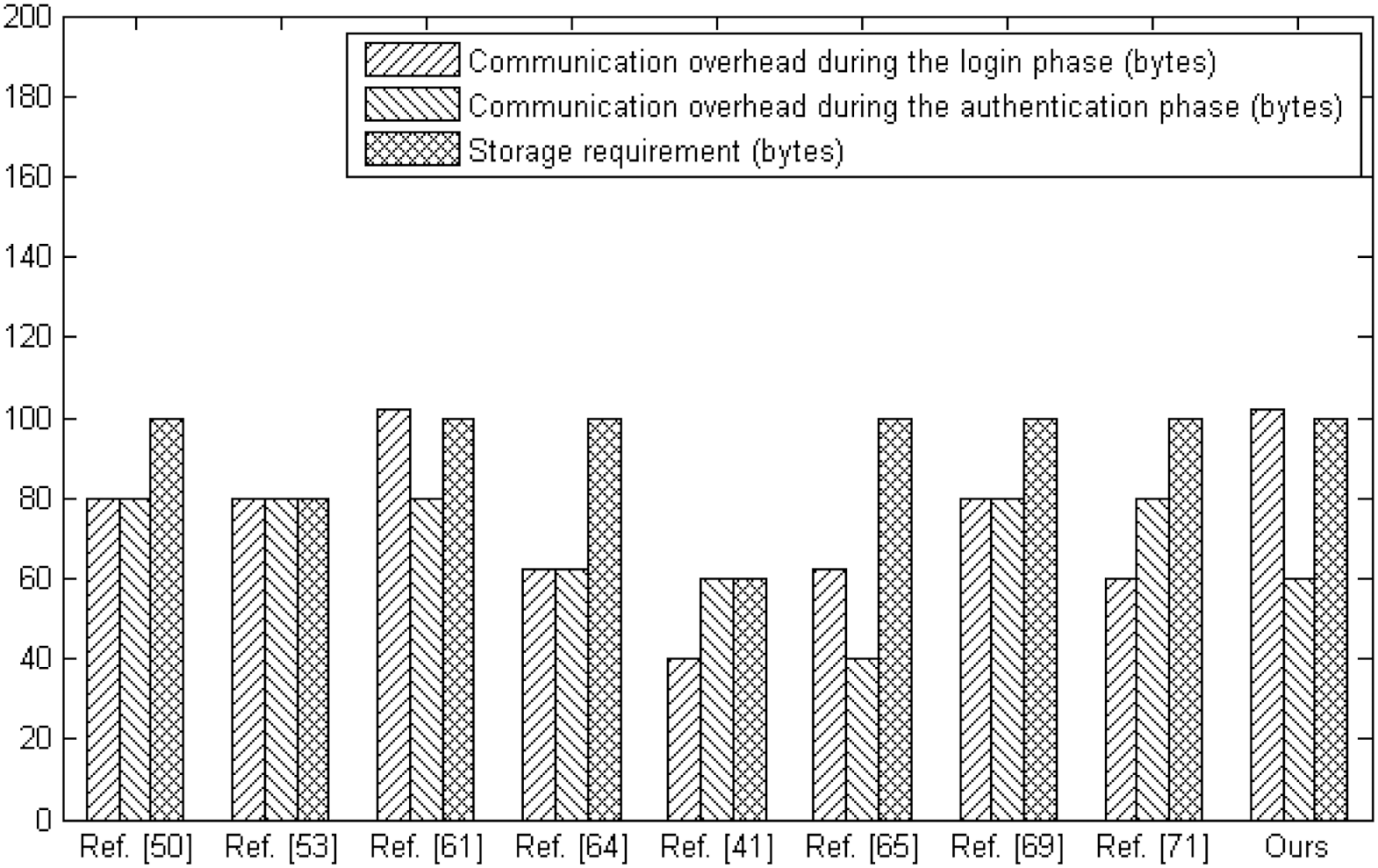
The communication overhead and storage requirement comparison.

Furthermore, Table 8 and Fig 7 show the comparisons regarding on communication overhead and storage requirement. Similarly, we adopt some following notations in the Table 8, where S1 represents the communication overhead during the login phase, S2 represents the communication overhead during the authentication phase, S3 represents the total communication overhead and S4 represents the storage requirement. With the same level of storage requirement, our protocol shows a satisfactory performance on the communication overhead.

**Table 8:** The communication overhead and storage requirement comparison.

	Ref. [50]	Ref. [53]	Ref. [61]	Ref. [64]	Ref. [41]	Ref. [65]	Ref. [69]	Ref. [71]	Ours
S1	80 bytes	80 bytes	102 bytes	62 bytes	40 bytes	62 bytes	80 bytes	60 bytes	102 bytes
S2	80 bytes	80 bytes	80 bytes	62 bytes	60 bytes	40 bytes	80 bytes	80 bytes	60 bytes
S3	160 bytes	160 bytes	182 bytes	124 bytes	100 bytes	102 bytes	160 bytes	140 bytes	162 bytes
S4	100 bytes	80 bytes	100 bytes	100 bytes	60 bytes	100 bytes	100 bytes	100 bytes	100 bytes

Both Reddy et al. [69] and Irshad et al. [71] who proposed other improvements of Wang et al.’s scheme also have done well jobs. In this sense, we are in the same field with these groups. However, there are notable characters to distinguish our work. After the cryptanalysis of Wang et al.’s scheme, we have applied novel methods to remedy their weaknesses, which is not included in other improved schemes. For example, we have adopted new ways to resist the user impersonation attack, privileged insider attack and server spoofing attack, and provide the perfect forward secrecy, respectively. Furthermore, our work is focus on reducing the computational complexity and providing more functionalities in a distinct way. In particular, compared with other improved works, our scheme has obvious advantages in the computational complexity with the same level of communication overhead and storage requirement.

## Conclusion

This paper cryptanalyzes Wang et al.’s scheme. In particular, we indicate that their protocol is still vulnerable to the user impersonation attack, privileged insider attack and server spoofing attack. Furthermore, their protocol fails to provide the perfect forward secrecy. As a remedy of these aforementioned problems, we propose a biometrics-based authentication and key agreement scheme for multi-server environments. Our protocol improves Wang et al.’s scheme. Discussions relating to security, functionality and efficiency are performed. Furthermore, results show that the proposed scheme satisfies these requirements mentioned above. Compared with other related schemes, our protocol achieves the stronger security and provides more functionality properties. Besides, the presented scheme requires the lower computational cost and shows a satisfactory performance on the communication overhead with the same level of storage requirement. Thus, the proposed protocol is suitable for expert systems and other multi-server architectures, such as, on-line medicine systems, on-line shopping systems and so on. Consequently, we conclude that our protocol is more appropriate in the multi-server environments.
